# Targeted expression profiling reveals distinct stages of early canine fibroblast reprogramming are regulated by 2-oxoglutarate hydroxylases

**DOI:** 10.1186/s13287-020-02047-1

**Published:** 2020-12-09

**Authors:** Ian C. Tobias, Mian-Mian C. Kao, Thomas Parmentier, Hailey Hunter, Jonathan LaMarre, Dean H. Betts

**Affiliations:** 1 Department of Physiology and Pharmacology, Schulich School of Medicine & Dentistry, Western University, Dental Sciences Building, Room DSB 2022, London, Ontario N6A 5C1 Canada; 2 Present Affiliation: Department of Cell and Systems Biology, University of Toronto, Toronto, Ontario Canada; 3 Department of Biomedical Sciences, University of Guelph, Guelph, Ontario Canada; 4 Children’s Health Research Institute, Lawson Health Research Institute, London, Ontario Canada

**Keywords:** Reprogramming, Pluripotent, Mesenchymal-to-epithelial transition, Canine, DNA hydroxymethylation, TET dioxygenase, 2-Oxoglutarate-dependent hydroxylase, Ascorbic acid, Retinoic acid, Embryonic stem cell

## Abstract

**Background:**

Ectopic expression of a defined set of transcription factors allows the reprogramming of mammalian somatic cells to pluripotency. Despite continuous progress in primate and rodent reprogramming, limited attention has been paid to cell reprogramming in domestic and companion species. Previous studies attempting to reprogram canine cells have mostly assessed a small number of presumptive canine induced pluripotent stem cell (iPSC) lines for generic pluripotency attributes. However, why canine cell reprogramming remains extremely inefficient is poorly understood.

**Methods:**

To better characterize the initial steps of pluripotency induction in canine somatic cells, we optimized an experimental system where canine fetal fibroblasts (cFFs) are transduced with the Yamanaka reprogramming factors by Sendai virus vectors. We use quantitative PCR arrays to measure the expression of 80 target genes at various stages of canine cell reprogramming. We ask how cFF reprogramming is influenced by small molecules affecting the epigenomic modification 5-hydroxymethylcytosine, specifically L-ascorbic acid and retinoic acid (AA/RA).

**Results:**

We found that the expression and catalytic output of a class of 2-oxoglutarate-dependent (2-OG) hydroxylases, known as ten-eleven translocation (TET) enzymes, can be modulated in canine cells treated with AA/RA. We further show that AA/RA treatment induces TET1 expression and facilitates early canine reprogramming, evidenced by upregulation of epithelial and pluripotency markers. Using a chemical inhibitor of 2-OG hydroxylases, we demonstrate that 2-OG hydroxylase activity regulates the expression of a subset of genes involved in mesenchymal-to-epithelial transition (MET) and pluripotency in early canine reprogramming. We identify a set of transcription factors depleted in maturing reprogramming intermediates compared to pluripotent canine embryonic stem cells.

**Conclusions:**

Our findings highlight 2-OG hydroxylases have evolutionarily conserved and divergent functions regulating the early reprogramming of canine somatic cells and show reprogramming conditions can be rationally optimized for the generation of maturing canine iPSC.

## Background

Domestic dogs are susceptible to a variety of inherited and acquired diseases with analogous conditions in human patients [1, 2]. Multiple factors have likely contributed to these shared pathologies including exposure to common environmental mutagens and toxins during human-canine coexistence [3], as well as similarities in immune system anatomy and development [4]. Due to these clinical similarities and even shared genetic etiology identified with comparative genomics, the dog has emerged as a powerful model to understand human genetic diseases [5–7]. Modeling the effect of disease-associated genetic variants in an assortment of cell types has been made feasible by the overexpression of a defined set of factors to generate induced pluripotent stem cells (iPSCs) [8, 9]. Ectopic expression of human or murine orthologues of the transcription factors OCT4, SOX2, KLF4, and MYC (OSKM) have been reported to generate putative canine iPSC lines [10–13]. However, the routine use of canine iPSCs has been limited by a lack of standardized canine reprogramming protocols and has led to inconsistent use of various reagents and donor somatic cells [14]. Moreover, few clonal canine iPSC lines have been established [15, 16] and no quantitative evidence of long-term canine iPSC self-renewal has been reported. Studies of several putative canine iPSC lines have revealed unique features compared to human iPSC such as the requirement of exogenous leukemia inhibitor factor (LIF) in addition to fibroblast growth factor 2 (FGF2) for proliferation in the undifferentiated state [11, 15]. Reprogramming canine fibroblasts with integrating vectors is reported to be a highly protracted process, ranging from 30 to 40 days [17]. Yet the molecular events during canine reprogramming have been neither characterized nor temporally defined. An understanding of the bottlenecks in canine somatic cell reprogramming is critical to improving the probability of successful reprogramming and mapping the molecular events during canine pluripotency acquisition.

As reprogramming cell transition towards the induced pluripotent state, distinct molecular events occur at defined timepoints that broadly segment reprogramming into initiation, maturation, and stabilization phases [18–20]. Global analyses of epigenetic modifications in reprogramming intermediates suggest that direct reprogramming to pluripotency is constrained by nuclear determinants controlling the redistribution of repressive chromatin modifications [21, 22]. iPSC derivation experiments and computational modeling indicate reprogramming transcription factors bind previously silent regulatory sequences to facilitate local epigenetic remodeling and activate gene transcription, including critical pluripotency regulators [23, 24]. Targeted genomic DNA demethylation during iPSC generation and maintenance of hypomethylated chromatin in pluripotent cells are mediated by the ten-eleven translocation (TET) family of methylcytosine dioxygenase enzymes [25, 26]. TET enzymes require 2-oxoglutarate (2-OG) to mediate DNA demethylation through the oxidation of 5-methylcytosine (5-mC) into a variety of cytosine substituents, which are then recognized by base-excision repair machinery [27, 28].

The longest-lived product of TET catalysis is 5-hydroxymethylcytosine (5-hmC) [29]. 5-hmC shows genome-wide overlap with OCT4 and NANOG motifs as well as histone modifications indicative of regulatory elements [30]. TET recruitment by reprogramming factors or by indirect effectors are thought to lead to active demethylation, particularly at enhancers and promoters, prior to chromatin opening and transcriptional activation [31]. At least one competent TET paralogue is required for the successful acquisition of pluripotency by mouse somatic cell reprogramming [32]. The importance of active demethylation is underscored in a mouse embryonic fibroblast (MEF) reprogramming model lacking the base-excision repair pathway component thymine DNA glycosylase, rendering cells incapable of reprogramming due to an impediment to epigenetic activation of miRNA clusters crucial to mesenchymal-to-epithelial transition (MET) [32].

Several endogenously produced small molecules regulate TET abundance, catalytic activity, and/or recruitment such as alpha-ketoglutarate [33], L-ascorbic acid (vitamin C) [34, 35], and retinoic acid (a derivative of vitamin A) [36, 37]. L-ascorbic acid has been reported to improve the kinetics of both mouse and human somatic cell reprogramming, which suggests an evolutionarily conserved role in direct reprogramming to pluripotency [38, 39]. Moreover, retinoic acid signaling is required for reprogramming to a naïve pluripotent state in mice [40]. It is conceivable that TET proteins may participate in active DNA demethylation, or otherwise enable more permissive histone modifications by cross-regulatory mechanisms, at regulatory elements for key determinants of pluripotency during canine somatic cell reprogramming. However, the soluble factors controlling TET abundance and activity in a somatic nuclear environment and how they could be leveraged in reprogramming canine cells have yet to be explored. Taking advantage of the non-integrating CytoTune-iPS 2.0 Sendai virus gene-delivery system, which are designed as self-limiting and non-transmissible [41, 42], we investigated whether modifiers of DNA methylation could promote early canine reprogramming. We highlight that initiation of canine fetal fibroblast reprogramming exhibits features of MET. Both the MET-like transcriptional response and early pluripotency gene upregulation can be bolstered by L-ascorbic acid and retinoic acid at concentrations that enhance TET-mediated 5-hmC generation. Although barriers to canine iPSC maturation still exist, we define a subset of pluripotency-associated transcription factors enriched at the transcript level in canine embryonic stem cells (cESCs) that may promote the acquisition of stable pluripotency in canine cells.

## Materials and methods

### Fibroblast and embryonic stem cell culture

Cryovials of primary canine adult fibroblasts at passage 2 (T0269) were purchased from Applied Biological Materials Inc. (Vancouver, Canada). Additionally, cryovials of passage 1 or 2 canine fetal fibroblasts derived from mixed-breed beagles were a gift from Dr. Jeong Yeon-Woo of Sooam Biotech Research Foundation (Seoul, South Korea) [43]. Adult or fetal canine fibroblasts were maintained in high-glucose DMEM supplemented with 10% Gibco™ qualified, Canada-origin fetal bovine serum (FBS), 2 mM GlutaMax, 1X non-essential amino acids, and 0.1 mM 2-mercaptoethanol. MEF monolayer preparation and culture of canine ESCs were conducted as previously described [44, 45]. Briefly, E12.5 DR4 MEFs obtained from Applied StemCell (CA, USA) were mitotically arrested by γ-irradiation and seeded at 1.5 × 10^4^ cells/cm^2^ for ESC co-culture. Canine ESC (cESC) lines derived at the Ontario Veterinary College from embryo explants (EX2, EX5, and EX7) were seeded onto γ-irradiated MEFs in a medium composed of KnockOut DMEM/F12, 15% KnockOut Serum Replacement, 1X GlutaMax, 1X non-essential amino acids, 0.1 mM 2-mercaptoethanol, 10 ng/mL human LIF, and 4 ng/mL human FGF2. Maintenance cultures of cESCs were propagated by picking and mechanically dissociating individual colonies into fragments. Canine partially reprogrammed (cPR) cell lines were maintained in MEF-conditioned cESC medium and split every 4–5 days with TrypLE Express enzyme. Incubators were maintained at 37 °C, 5% CO_2,_ and ambient oxygen. Unless otherwise stated, all cell culture reagents were obtained from Thermo Fisher Scientific.

### Somatic cell reprogramming

Two days prior to transduction, canine fibroblasts were seeded at 5 × 10^4^ cells per well of a six-well plate. Using the CytoTune-iPS 2.0 Reprogramming Kit (Thermo Fisher Scientific), Sendai viral vectors (SeV) encoding human KOS (KLF4/OCT4/SOX2), c-MYC, and KLF4 were added to individual wells containing 2–2.5 × 10^5^ cells according to the manufacturer’s protocol with some modifications. The multiplicity of infection (MOI) for KOS and c-MYC SeV vectors were adjusted to six (an average of six viral particles per cell) and the KLF4-SeV vector MOI was increased from three to five, based on transduction efficiencies achieved using CytoTune emGFP reporter (Thermo Fisher Scientific).

For experimental manipulation of early canine reprogramming, fibroblast medium was supplemented with 100 μM ascorbic acid and 0.1 nM retinoic acid (AA/RA) or an equivalent volume of diluent (vehicle) beginning 2 days post-infection (DPI). Treatments were applied with daily medium exchanges for 4 days. At 6 DPI, the transduced cells were bulk passaged and transferred (3–5 × 10^4^ cells) to 10-cm culture dishes containing 2 × 10^4^/cm^2^ γ-irradiated CF1 MEFs (Applied Stem Cell). For analysis of 6 DPI intermediates, 0.8 × 10^6^ cells were pelleted, submerged in RNAlater (Sigma Aldrich), and stored at − 80 °C until nucleic acid extraction. Presumptive canine iPSC colonies were maintained in basal medium containing KnockOut DMEM/F12 containing 15% KnockOut Serum Replacement (KOSR) [45, 46] or high-glucose DMEM with 10% FBS. Co-cultures of transduced canine fibroblasts and γ-irradiated MEFs were visually monitored for focal epithelialization and the emergence of primary colonies. For clonal isolation (10–20 DPI), primary colonies were mechanically isolated with a pipet tip and transferred into Geltrex- and MEF-coated (25,000 MEF/well) 48-well plates. For analysis of 10 DPI intermediates, 50–70 primary colonies were mechanically isolated by with a pipet tip and submerged in RNAlater for storage at − 80 °C.

### RT-qPCR and quantification of relative transcript abundance

Total RNA from cultured cells was isolated using PureLink RNA kit (Thermo Fisher) and quantified using a NanoDrop spectrophotometer. One microgram of total RNA was treated with RQ1 DNase (Promega) and used to synthesize cDNA using the Superscript II Reverse Transcription kit (Thermo Fisher) with 1.25 μM each of oligo dT and random hexamers. qPCR reaction mixtures contained 5-μL SensiFast SYBR (BioLine), 1 μL primer mixture (500 nm final), and 2 μL cDNA template. PCR assays were run on using the CFX384 Real-Time System (BIO-RAD). Annealing temperatures were optimized for the amplification of a single product of expected size, excised, and sequenced at the London Regional Genomics Center to confirm specificity. All custom primer sequences and annealing temperatures can be found in Supplemental Table 1. Reaction data were corrected for individual primer amplification efficiencies, calculated from a dilution series standard. The expression ratio for genes of interest was normalized to two reference genes (TBP and RPS5) and calculated using the delta-delta Ct method.

For the RT^2^ Profiler PCR array, 400 ng of DNase-treated RNA was reverse transcribed to cDNA using the RT^2^ First Strand Kit (Qiagen). Quantitative PCR was performed using the Canine Epithelial-Mesenchymal Transcription RT^2^ Profiler PCR Array (Qiagen) according to the manufacturer’s guidelines. Alternatively, the expression of the canine homologues of reprogramming-associated genes were measured with a custom RT^2^ Profiler PCR Array (Qiagen). All transcripts targeted with the custom RT^2^ Profiler PCR Array are listed in Supplemental Table 2. Data generated from the qPCR arrays was analyzed as expression ratios using sets of five reference genes. For the RT^2^ Profiler PCR Array Dog Epithelial-Mesenchymal Transition (PAFD-090Z), the reference genes used were ACTB, B2M, GAPDH, HPRT1, and RPLP1. For the RT^2^ Profiler PCR Array Custom Dog Reprogramming (CAPF14050), the reference genes used were GAPDH, HPRT1, RPLP1, PPIA, and TBP. Among transcripts targeted by the custom dog reprogramming PCR array, only LIN28B, NR5A2, and LEFTY1 were not detected and excluded from further analysis.

### Targeted bisulfite-PCR and promoter methylation analysis

Genomic DNA from cultured cells was isolated using GenElute Mammalian Genomic DNA Miniprep kit (Sigma Aldrich). Genomic DNA was eluted in nuclease-free water, and sample concentrations were determined using NanoDrop spectroscopy (Thermo Fisher Scientific). Bisulfite modification and purification was performed using the EZ DNA Methylation Gold kit (Zymo Research) with 400 ng of genomic DNA. Bisulfite-converted DNA was used immediately for PCR using Platinum Taq Polymerase and primer pairs designed to target the 5′-flanking sequences close to the transcriptional start site of canine POU5F1 and NANOG [15] (Supplemental Table 3). Nested re-PCR was performed with 3 μL of the resultant bisulfite-PCR reaction mixture. PCR amplifications used variations of the following thermal cycling protocol: 3 min at 94 °C; 35 cycles of 94 °C for 20 s, 59–64 °C gradient for 15 s, 72 °C for 15 s; 72 °C for 5 min final extension. In nested re-PCR reactions, a touchdown annealing range of 70–64 °C was added before amplification cycles.

PCR amplicons were ligated into pCR2.1 plasmids and transformed into competent *E. coli* using the TA cloning kit (Thermo Fisher). Bacteria were spread onto LB agar and grown overnight in a 37 °C bacterial incubator. Clones were picked, transferred into LB medium with ampicillin, and grown in suspension for 16 h. The plasmid DNA were purified with Presto Mini Plasmid kit (Frogga Bio) and sequenced at the London Regional Genomics Centre (Robarts Research Institute, London, ON) with 3730 DNA Analyzer System (Applied Biosciences). Base calls, quality trimming, and vector clipping were performed using pre-Gap and Gap4 (Staden Package) [47]. To determine accurate methylation patterns on target regions, at least 10 processed clone sequences were aligned to the reference genomic sequence for each biological replicate using the R/Bioconductor package methVisual [48]. The methylation state for each CpG site was exported from the quality-checked aligned sample sequences. For each acceptable clone, we adjusted the cytosine methylation level based on the bisulfite conversion rate (Bis_con_) with each bisulfite-PCR reaction:

$$ \mathrm{Adjusted}\ \mathrm{Cytosine}\ \mathrm{Methylation}\ \mathrm{Level}=\frac{\left(\raisebox{1ex}{${C}_{\mathrm{meth}}$}\!\left/ \!\raisebox{-1ex}{${\mathrm{Bis}}_{\mathrm{con}}$}\right.\right)}{C_{\mathrm{total}}} $$

### Immunolabeling for fluorescence microscopy and flow cytometry

For fluorescence microscopy, cells were stained for 1 h with CD44-PE (IM7; 12-0441-82; 1:100 dilution) or CD90-PE (YKIX337.217; 12-5900-42; 1:50 dilution) diluted in a 3% BSA staining solution. Cells were imaged on the stage of a Leica DMI 6000B microscope. Digital images were captured with an Orca Flash camera (Hamamatsu Photonics) and Application Suite X software (Leica Microsystems). Brightness and contrast were standardized to isotype stained samples, and the equivalent imaging parameters were applied to all other images using ImageJ (National Institutes of Health, MD).

For flow cytometry, cells were first collected by a cell scraper after treating with enzyme-free cell dissociation buffer containing EDTA for 5 min and further dissociated with TrypLE enzyme for 6 min at 37 °C. Single live cells in suspension were filtered through a 70-μm strainer adjusted to a density of approximately 10^6^ cells/mL in staining buffer (1% bovine serum albumin, 0.075% sodium azide) and incubated with CD90-PE (1:50 dilution), CD44-PE (1:100 dilution), or an isotype control for 1 h protected from light. Cells were washed twice with staining buffer and stained with 7-aminoactinomycin D reagent to exclude dead cells. Events were recorded using the Accuri C6 platform (BD Bioscience) using the standard laser configuration and the following optical filters: 533/30, 585/40, 670 LP, and 675/25. Single color or fluorescence minus one control samples containing an equivalent volume of diluent were included to set analysis gates and to threshold background autofluorescence. Samples stained with the IgG isotype control (12-4031-82) were used to determine non-specific antibody binding. All antibodies were obtained from Thermo Fisher Scientific.

### 5-Methylcytosine and 5-hydroxymethylcytosine quantification

Genomic DNA was isolated as described above (see the “Targeted bisulfite-PCR and promoter methylation analysis” section), aliquoted, and diluted to 10 ng/μL. Global 5-methylcytosine or 5-hydroxymethylcytosine levels were assessed from 75 to 150 ng of total gDNA using 5-mC DNA ELISA Kit or Quest 5-hmC DNA ELISA Kit (Zymo Research), according to the manufacturers’ recommendations. Briefly, percent 5-mC or 5-hmC was interpolated from a five-point standard curve and adjusted based on the CpG dinucleotide density of the reference genome assembly for the species under investigation.

### Statistical analysis

Sample sizes and statistical method specific to each figure are denoted in figure legends. All statistical analyses and plotting were performed using R statistical software (version 3.5.0, The R Foundation for Statistical Computing, Vienna, Austria). A *P* value less than 0.05 was considered statistically significant. Experiments were not randomized, and no statistical method was used to predetermine sample size. When two groups were compared, an unpaired *t* test or, if assumptions of normality and homoscedasticity were violated, a Mann-Whitney test was performed. For multiple group comparisons, either one-way analyses of variance (ANOVA) or the non-parametric Welch’s ANOVA was applied. A two-way ANOVA was performed on datasets with two grouping variables. Where significance existed for a grouping factor, Tukey or Dunn post hoc methods were carried out to determine significant differences between group means. For correlation analyses, the linear association between RT^2^ Profiler qPCR array expression datasets was computed using the non-parametric Kendall rank correlation coefficient. Principal component analysis was performed using the PCAtools package, and heatmaps were generated using the pheatmap package [49, 50].

## Results

### Adult canine fibroblasts are refractory to transcription factor-mediated reprogramming

Limited progress has been reported in canine cellular reprogramming, in contrast to the rapid acceleration of iPSC technologies in primates and rodents. Moreover, the contribution of numerous cellular pathways or molecular processes, such as DNA methylation, as barriers to canine pluripotency induction and maintenance has yet to be explored. We established canine partially reprogrammed (cPR) cell lines in a previous attempt to reprogram canine adult fibroblasts (cAFs) with the CytoTune-iPS Sendai vectors. Interestingly, cPR lines could be scaled for greater than 20 passages in feeder-free culture with MEF-conditioned cESC medium (KOSR/LIF/FGF2) permitting the derivation of clonal lines from independent transductions. However, the cPR lines produce heterogeneous cultures consisting of colonies with compact centers and include cells positive for SSEA4, a marker of pluripotent cESCs [45], surrounded by cells with bipolar morphology (Supplemental Figure 1A). We explored to what extent cPR clones achieve promoter demethylation and expression of core pluripotency transcription factors. RT-qPCR analyses show that cPR clones have greater expression of the canine POUF51/OCT4 homologue compared to adult fibroblasts (Supplemental Figure 1B, *P* < 8.82 × 10^−3^). However, the transcript abundances for NANOG were not different compared to adult fibroblasts (*P* > 0.61), and SOX2 was only significantly upregulated in the cPR-L6 line (Supplemental Figure 1B, *P* = 9.81 × 10^−4^).

7To investigate the extent of pluripotency gene silencing, the methylation of the status of the promoter regions of canine NANOG and POU5F1 was assessed in cPR cells and parental cAFs compared to cESCs. Different CpG methylation profiles were observed in the NANOG promoter and the POU5F1 promoter between cAF, cPR, and primed type (LIF-FGF2) cESCs (Supplemental Figure 1C). The overall methylation level of the NANOG promoter region was lower in cESC compared to both cAF (*P* = 1.25 × 10^−3^) and cPR (*P* = 6.36 × 10^−4^) (Supplemental Figure 1D). Additionally, the degree of POU5F1 promoter methylation is lower in cESC compared to cAF (*P* = 0.032), but not cPR cells (*P* = 0.082) (Supplemental Figure 1E). These findings suggest that the triad of core pluripotency transcription factors are incompletely expressed in cPR clones. Moreover, cAFs were observed to be refractory to reprogramming by exogenous transcription factors as only partially reprogrammed clones exhibiting a methylated NANOG promoter could be attained.

### Canine fetal fibroblasts have greater TET expression, 5-hmC level, and proliferation rate compared to adult fibroblasts

**Fig. 1:**
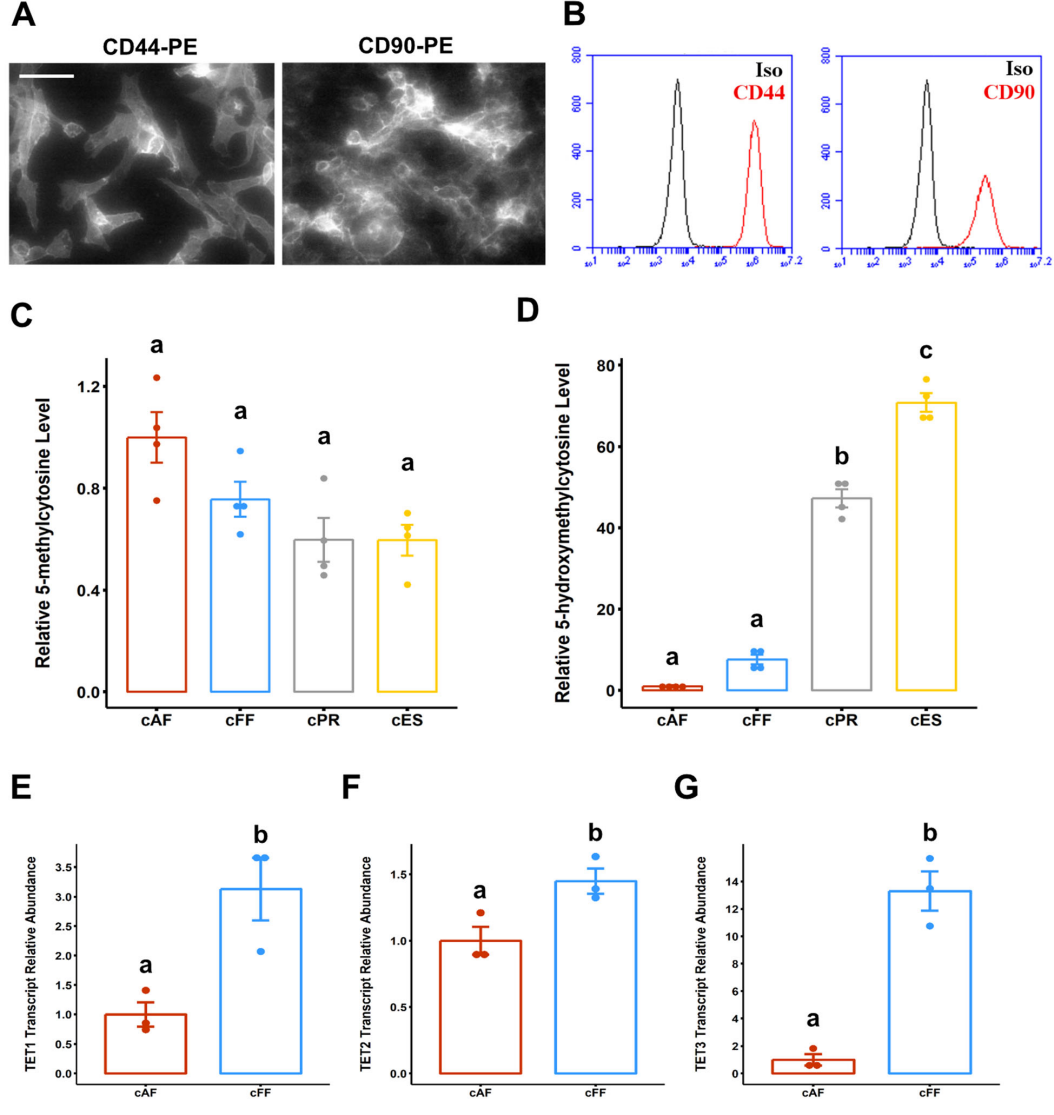
Cell type-dependent levels of 5-methylcytosine and 5-hydroxymethylcytosine. **a** Representative fluorescent micrographs for canine fetal fibroblasts (cFFs) stained with phycoerythin (PE)-conjugated antibodies against CD44 and CD90/THY1. Scale bar unit length is 50 μm. **b** Overlay histograms showing cFFs stained with PE-conjugated antibodies (red) or isotype control antibodies (black). Fold difference in **c** 5-methylcytosine and **d** 5-hydroxymethylcyctosine levels in canine adult fibroblast (cAF), canine fetal fibroblast (cFF), canine partially reprogrammed (cPR), and canine embryonic stem cells (cESCs). Data are presented as mean ± standard error, *n* = 4. Relative transcript abundance for **e** TET1, **f** TET2, and **g** TET3 in steady-state cultures of cAF and cFF. Data are presented as mean ± standard error, *n* = 3. Means annotated with different letters are considered significantly different by one-way analysis of variance and Tukey’s honestly squared difference test, *P* < 0.05

We reasoned that primary canine fibroblasts derived from less mature donor tissues may undergo reprogramming towards pluripotency more efficiently, which has been linked to both proliferative capacity and various nuclear determinants of the undifferentiated state [51, 52]. Canine fetal fibroblasts (cFFs) reported to be permissive to somatic cell nuclear transfer-mediated reprogramming [43] were assessed for their suitability as donor cells for OSKM transcription factor-mediated reprogramming. Fluorescent immunocytochemistry and flow cytometry revealed positive staining for mesenchymal surface antigens CD90/THY1 and CD44 [53, 54] on greater than 98% cultured cFFs (Fig. 1A, B, Supplemental Figure 2A). Early passage cFF cells also exhibited a more rapid proliferation rate compared to early passage cAFs (Supplemental Figure 2B-C, *P* = 0.038). Manipulation of genomic DNA methylation via de novo methyltransferase or TET dioxygenase enzymes have been effective strategies to increase the probability of complete reprogramming in both rodent and primate cells [55–57]. We analyzed both 5-mC and 5-hmC levels by DNA ELISA to determine if the degree of global cytosine methylation or cytosine hydroxymethylation differs among primary fibroblasts from adult or fetal donor tissues, cPR cells, and pluripotent cESCs. We observed that the percent of 5-methylcytosine did not significantly differ across cAF, cFFs, cPR cells, or cESCs (Fig. 1C). Basal 5-hmC was not significantly different between cFF and cAF (*P* = 0.98), and both fibroblast populations exhibit lower 5-hmC (~ 150-fold) compared to cESCs (*P* < 1 × 10^−7^) and cPR cells (Fig. 1D, *P* < 1 × 10^−7^). However, relative transcript abundance was significantly greater for the canine TET1 (Fig. 1E, *P* = 4.2 × 10^−2^), TET2 (Fig. 1F, *P* = 3.4 × 10^−2^), and TET3 (Fig. 1G, *P* = 9.0 × 10^−3^) in fetal compared to adult canine fibroblasts. These results suggest that canine somatic cells derived from less mature donor tissue exhibit greater abundances of TET paralogues, but maintain similar levels of cytosine modifications genome wide.

### Combined ascorbic acid and retinoic acid treatment facilitates mesenchymal-to-epithelial transition in early canine reprogramming

**Fig. 2:**
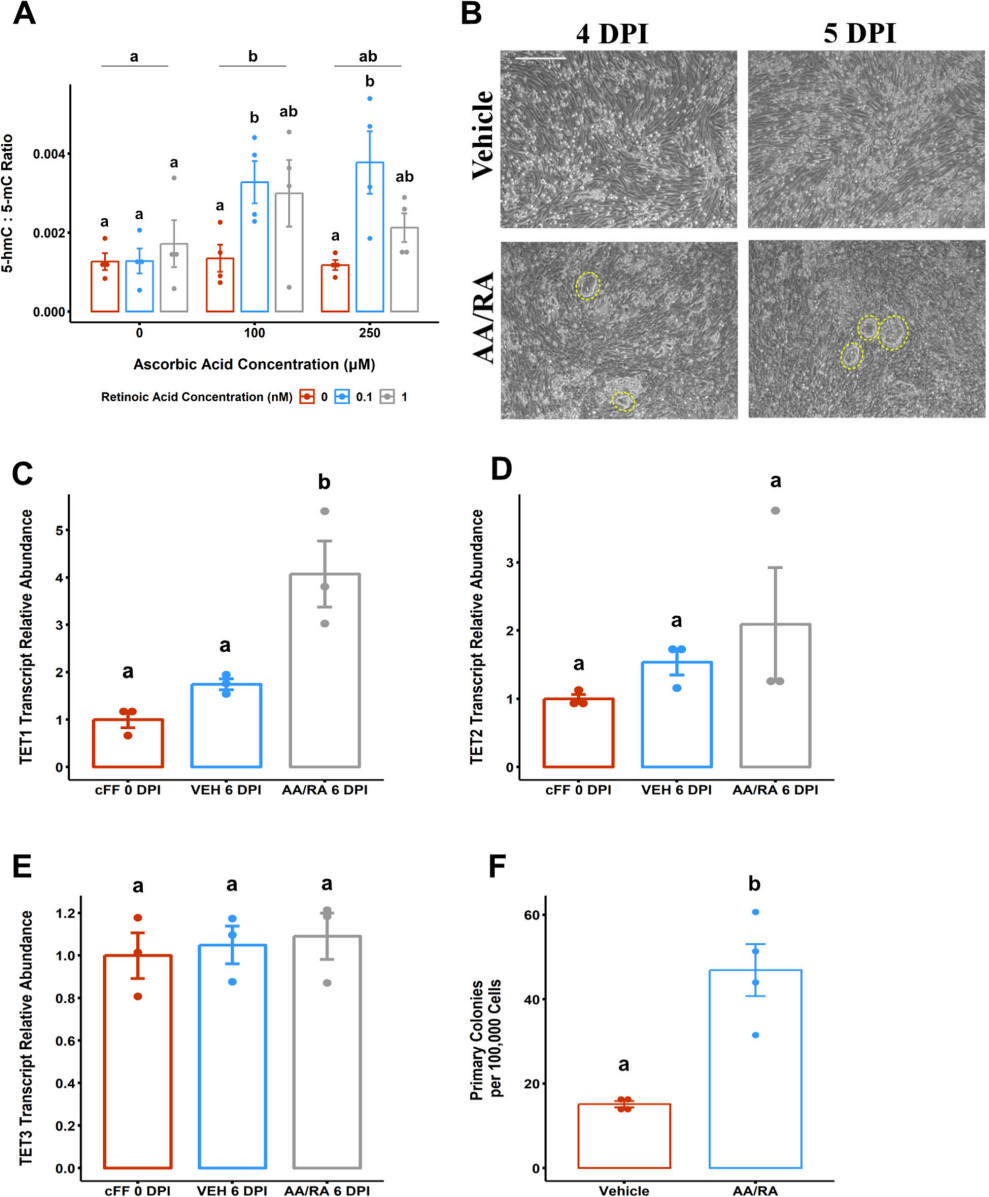
L-ascorbic acid and retinoic acid modulates 5-hmC level and promotes focal epithelialization in early canine reprogramming of TET paralogue expression. **a** 5-hmC to 5-mC ratio in canine fetal fibroblasts (cFFs) cultured in atmospheric oxygen and exposed to various concentrations of L-ascorbic acid (AA) and/or retinoic acid (RA). Each data point is calculated from paired aliquots from the same purified gDNA sample and analyzed by separate ELISA assay kits. **b** Representative phase-contrast micrographs of cFFs transduced with CytoTune-iPS 2.0 Sendai Reprogramming Kit (SeV 6:5:6) at 4 or 5 days post-infection (DPI). Transductants were treated with L-ascorbic acid and retinoic acid (AA/RA) or an equivalent volume of diluent (VEH). Yellow dashed outlines delimit areas of focal epithelial cluster formation exclusively in the AA/RA-treated transductants. Relative transcript abundance for **c** TET1, **d** TET2, and **e** TET3 in steady-state cFF, transduced cFF treated with VEH, or AA/RA. **F** Summarization of the primary colony counts (per 10^5^ transduced cells) performed 4 days after re-seeding on mouse embryonic fibroblast feeder cells (10 DPI). Data are presented as mean ± standard error, *n* = 3. Means annotated with different letters are considered significantly different by two-way analysis of variance or the two-sample Mann-Whitney test, *P* < 0.05. Scale bars are 250 μm

To investigate the role of canine TET paralogues in canine somatic cell reprogramming, we used small molecule supplementation as a strategy to manipulate reprogramming progression without genetic interventions. The enzymatic activity of TET enzymes is activated by exogenous cofactors or inhibited by structural analogues, both of which are broadly available and inexpensive [58]. We initially sought to profile pan-TET activity and TET paralogue expression in canine somatic cells upon exposure to combinations of RA and AA at concentrations applied to human or mouse iPSC generation [38, 59]. Using the ratio of genome-wide 5-hmC to 5-mC as an index of the overall catalytic output of TET enzymes [60], we observed both AA (Fig. 2A, *F* = 4.08, *P* = 2.83 × 10^−2^) and RA (Fig. 2A, *F* = 6.73, *P* = 4.26 × 10^−3^) have a significant independent contribution on the observed 5-hmC to 5-mC ratio in cFFs. Only 100 μM AA (Fig. 2A, *P* = 0.033) and 0.1 nM RA (Fig. 2A, *P* = 0.035) significantly elevated 5-hmC to 5-mC ratio compared to vehicle controls. Exposure of canine fibroblast to various RA concentrations did not alter TET paralogue expression (Supplemental Figure 2D-F), whereas significant changes in canine TET1 (Supplemental Figure 2D, *P* = 0.0171) and TET3 (Supplemental Figure 2F, *P* = 6.29 × 10^−3^) abundance were only detected at 250 μM AA conditions compared to vehicle control. Collectively, these results suggest that combinatorial exposure to 100 μM AA and 0.1 nM RA increases 5-hmC level relative to 5-mC, independent of changes to TET paralogue expression.

How genes associated with reprogramming to a pluripotent state are regulated in transcription factor-mediated reprogramming from canine somatic cells is still unknown. To fill this gap, we leveraged the non-integrating Sendai virus (SeV) reprogramming platform (CytoTune-iPS 2.0 Kit), designed to reprogram human somatic cells and reported to reprogram murine fibroblasts to deliver the human OSKM homologues to cFFs [41]. Based on transduction efficiencies observed with the control SeV-eGFP vector, we transduced cFF cultures at less than 20% confluency with SeV particles at multiplicity of infection (MOI) values of at least five (Supplemental Figure 2G-H). Only cFF transductants treated with AA/RA produced focal cell clusters with epithelial character by 4 DPI with observable increases in size and frequency by 5 to 6 DPI (Fig. 2B). Interestingly, we observe that AA/RA-treated cells exhibit greater expression of TET1 (Fig. 2C, *P* = 1.80 × 10^−2^), but neither TET2 (Fig. 2D, *P* = 0.72) nor TET3 (Fig. 2E, *P* = 0.96). In addition, reprogramming intermediates exposed to AA/RA generate primary colonies more frequently upon re-seeding onto MEF feeders compared to vehicle-treated controls (Fig. 2F, *P* = 2.86 × 10^−2^).

**Fig. 3:**
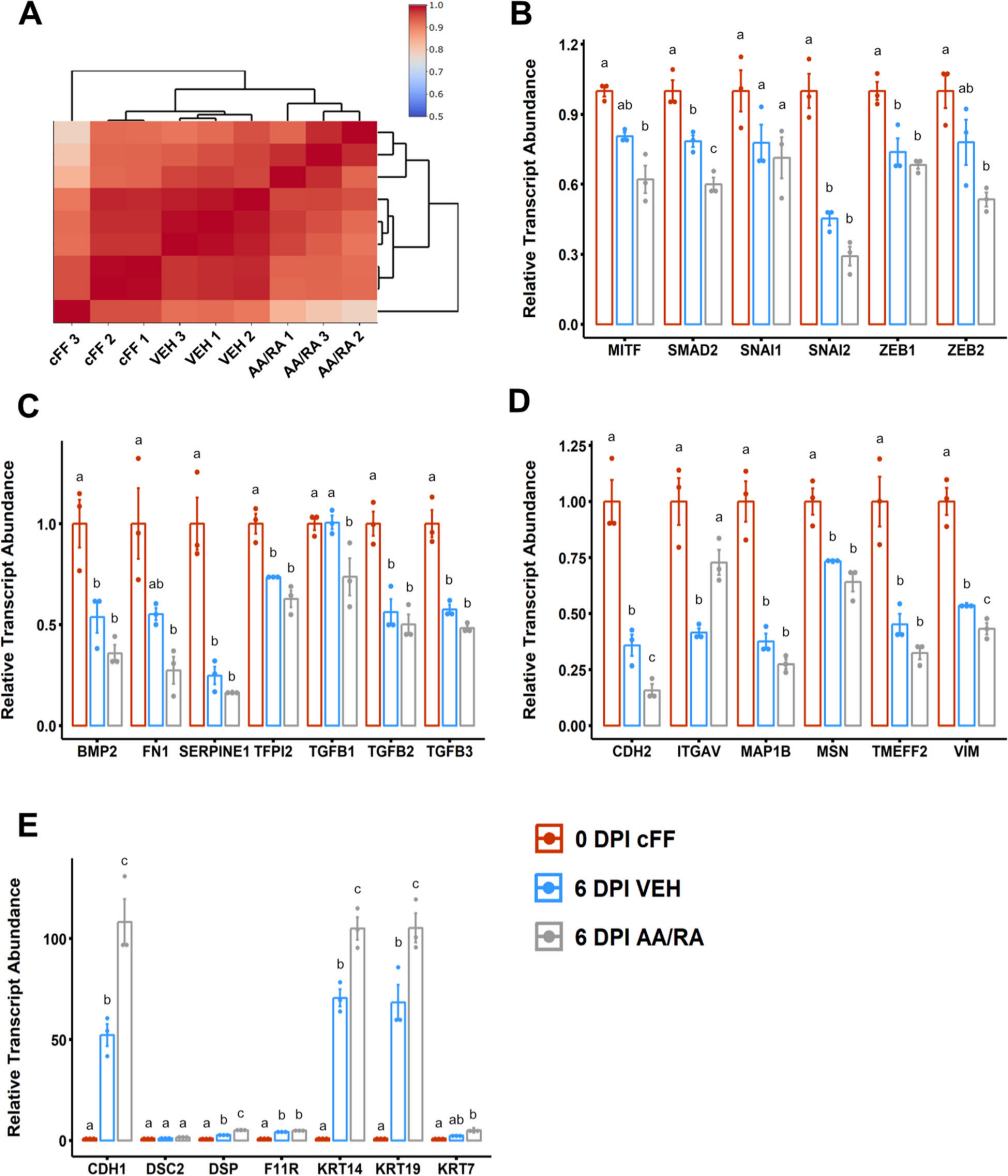
L-ascorbic acid and retinoic acid enhance a transcriptional response resembling mesenchymal-to-epithelial transition. **a** Heatmap of Pearson correlation coefficients of transcript panel abundances in each sample and grouped by hierarchical clustering. **b** Relative abundances of transcripts associated with regulation of gene expression. **c** Relative abundances of transcripts associated with secreted ligands or matrix components. **d** Relative abundances of transcripts associated with the cytoskeleton or cell adhesion that are downregulated at 6 DPI. **e** Relative abundances of transcripts associated with the cytoskeleton or cell adhesion that are upregulated at 6 DPI. Data are presented as mean ± standard error, *n* = 3. Means annotated with different letters are considered significantly different by one-way analysis of variance and Tukey’s honestly squared difference test, *P* < 0.05

We next examined the abundance of transcripts associated with mesenchymal or epithelial cell identity in 6 DPI reprogramming intermediates by the qPCR array. Correlation analysis and hierarchical clustering revealed that targeted expression profiles of biological replicates both grouped together and were highly similar (Kendall’s tau coefficient *τ* > 0.94), suggesting that merging data across arrays was robust to batch effects (Fig. 3A). This analysis also highlighted that AA/RA-treated intermediates were more dissimilar to non-transduced cFF cells than vehicle-treated intermediates at 6 DPI. The transcripts of several DNA-binding proteins involved in the repression of epithelial gene expression were downregulated in 6 DPI reprogramming intermediates compared to cFF cultures that did not receive SeV reprogramming factors (SNAI2, ZEB1) (Fig. 3B, *P* < 8.63 × 10^−3^). Furthermore, SMAD2 transcript was significantly decreased in AA/RA intermediates compared to transductants receiving only vehicle diluent (Fig. 3B, *P* = 1.08 × 10^−2^). Similarly, 6 DPI intermediates exhibited a lower abundance of several mesenchyme-associated transcripts that encode secreted proteins (FN1, BMP2, SERPINE1) compared to parental cFF cells (Fig. 3C, *P* = 1.99 × 10^−2^). Among three mammalian transforming growth factor beta (TGFβ) isoforms, only TGFβ1 was significantly decreased in AA/RA intermediates relative to vehicle control (Fig. 3C, *P* = 4.28 × 10^−2^). Strikingly, several transcripts related to cytoskeletal (MSN, MAP1B, KRT7) and plasma membrane (F11R, TMEFF2) components were differentially abundant in reprogramming intermediates compared to donor cFF cells (Fig. 3D, E, *P* < 1.30 × 10^−2^). Notably, mesodermal markers CDH2 (*P* = 4.51 × 10^−2^) and VIM (*P* = 3.55 × 10^−2^) were significantly lower in AA/RA-treated intermediates compared to the vehicle group (Fig. 3D), whereas hallmark transcripts of epithelial cell types including CDH1 (*P* = 2.31 × 10^−3^), DSP (*P* = 6.54 × 10^−3^), KRT14 (*P* = 2.43 × 10^−3^), and KRT19 (*P* = 1.72 × 10^−2^) were significantly elevated in AA/RA reprogramming intermediates compared to vehicle-treated transductants (Fig. 3E). Together, these findings indicate that AA/RA supplementation during the early reprogramming of canine fibroblasts can facilitate a transcriptional response resembling mesenchymal-to-epithelial transition (MET).

### Targeted expression profiling of canine reprogramming intermediates identifies genes controlled by 2-oxoglutarate-dependent hydroxylase activity

We asked if genes associated with canine cell reprogramming are regulated by 2-oxyglutarate-dependent (2-OG) hydroxylases including epigenetic modifiers such as the TET paralogues. The contribution of pan-TET enzymatic activity in AA/RA reprogramming conditions was assessed using dimethyloxalylglycine (DMOG), a competitive inhibitor of 2-OG hydroxylase activity [61, 62]. We titrated the concentration of DMOG in cFF culture medium and observed that genome-wide 5-hmC level decreased in a dose-dependent manner (Supplemental Figure 3A, *P* = 9.46 × 10^−6^). Both 100 μM and 300 μM DMOG decreased global 5-hmC (Supplemental Figure 3A, *P* = 1.58 × 10^−3^) and did not significantly affect population doubling intervals compared to vehicle-treated cFF (Supplemental Figure 3B, *P* = 0.57). Notably, reprogramming progression could not be reproducibly assessed by alkaline phosphatase staining due to high endogenous levels of the enzyme in bulk transductants and heterogenous colony staining at the 10 DPI timepoint (Supplemental Figure 3C-D).

**Fig. 4:**
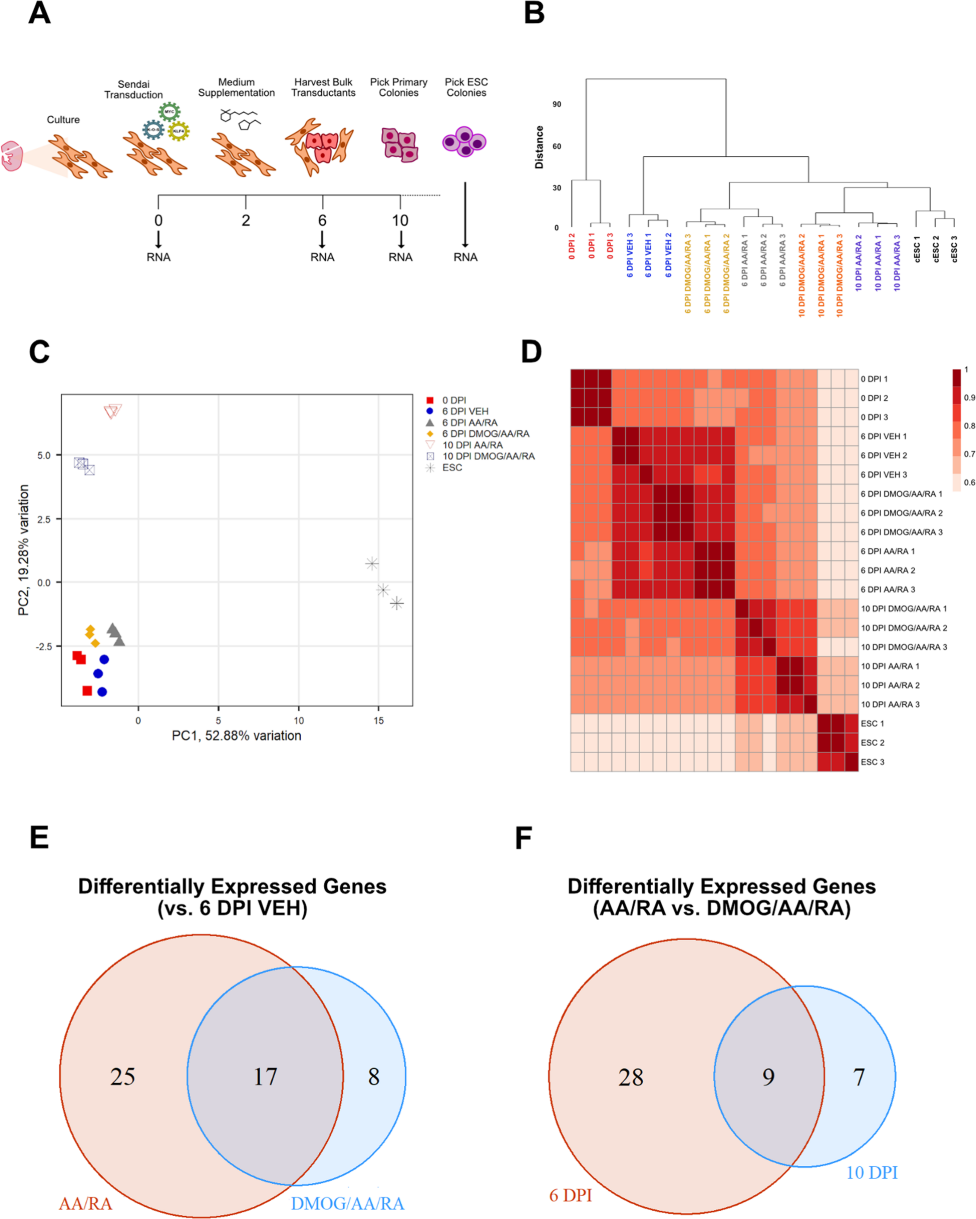
Exploratory data analysis and summarization of differential gene expression for targeted expression profiles of canine reprogramming intermediates. **a** Schematic of canine fetal fibroblast (cFF) reprogramming and timepoints of RNA sample collection. **b** Hierarchical clustering analysis of all samples based on gene expression. **c** Principal component analysis of all samples based on gene expression. **d** Correlation heatmap of all samples based on gene expression. Sequential color scale represents Kendall’s rank correlation coefficient. **e** Venn diagram showing the number of differentially expressed genes versus the 6 DPI vehicle (VEH) group for 6 DPI AA/RA (red) and 6 DPI DMOG/AA/RA (blue). **f** Venn diagram showing the number of differentially expressed genes for AA/RA versus the corresponding DMOG/AA/RA group at 6 DPI (red) and 10 DPI (blue)

To gain a view of transcriptional regulation in early canine reprogramming, we carried out targeted expression profiling of reprogramming-associated transcripts in three biological replicates of intermediate cell populations by qPCR arrays (Fig. 4a). Hierarchical clustering showed that 6 DPI transductants exposed to AA/RA are less similar to vehicle-treated cells than transductants with the DMOG/AA/RA treatment (Fig. 4b). Principal component analysis revealed that cFFs harvested at 0 DPI were different from the three groups of 6 DPI cells infected with the Yamanaka reprogramming factors (Fig. 4c). Among 6 DPI samples, AA/RA-treated reprogramming intermediates least resembled 0 DPI cFFs. Ten DPI reprogramming intermediates were different from samples collected at 6 DPI as well as pluripotent cESC cultures. Signaling proteins (STAT3, SMAD1, BMPR1A) and transcription factors (DPPA5, NANOG, SALL4) involved in early embryonic development contribute to PC1 (52.88% of variation), which distinguishes cESC samples from cFFs and reprogramming intermediates (Supplemental Figure 3E). Biological replicates correlated well (Kendall’s tau coefficient *τ* > 0.91), particularly the collection of 6 DPI samples representing heterogenous populations in the early phase of reprogramming (Fig. 4d). Compared to vehicle-treated 6 DPI transductants, we detected a greater number of differentially expressed genes in reprogramming intermediates receiving AA/RA than those concomitantly exposed to DMOG (42 vs. 25) (Fig. 4e). The influence of DMOG/AA/RA treatment, based on the number of differentially expressed genes compared to cells receiving only AA/RA, is more pronounced in 6 DPI transductants than in primary colonies harvested at 10 DPI (37 vs. 16) (Fig. 4f). Nevertheless, we observe an overlap of several DMOG-sensitive transcripts (CDC42, EZH2, IL6ST, NR0B1, STAT3, ST3GAL2, TGFB1) which remain differentially expressed in 10 DPI samples. These findings indicate that exposure to AA/RA for a defined period in early reprogramming alters the expression of numerous DNA-binding, chromatin-binding, and signaling factors associated with the acquisition of pluripotency.

**Fig. 5:**
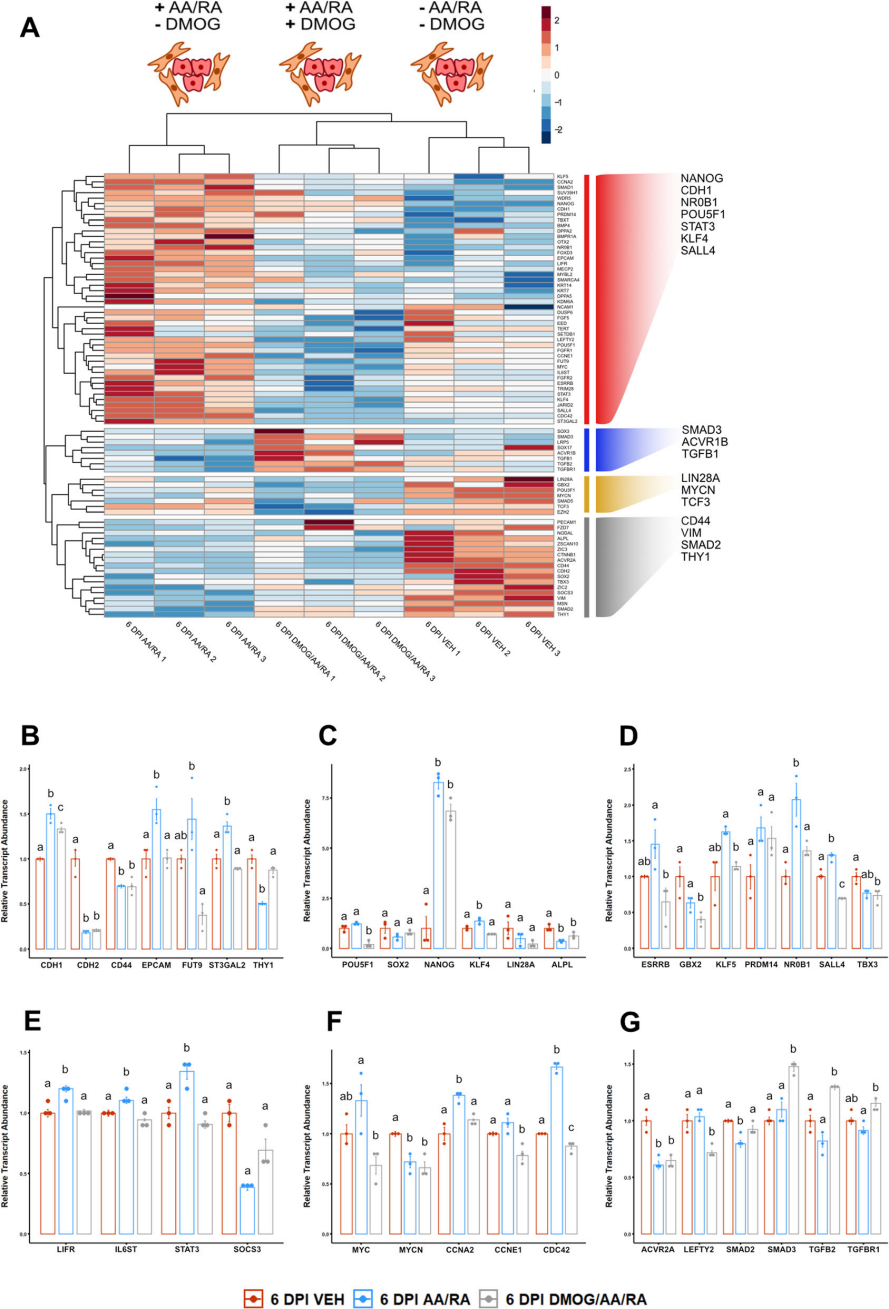
AA/RA medium supplementation in early canine reprogramming facilitates the upregulation of a subset of transcripts associated with pluripotency, proliferation, and LIF signaling. **a** Hierarchical cluster analysis to identify subsets of genes that show specific expression patterns across specific samples of early (6 DPI) canine reprogramming intermediates. Divergent color scale represents gene expression scaled by row. **b** Relative abundances of transcripts associated with cell surface proteins or the synthesis of cell surface antigens. **c** Relative abundances of transcripts associated with cell pluripotency. **d** Relative abundances of transcripts associated with early embryonic (naïve) pluripotency. **e** Relative abundances of transcripts associated with leukemia inhibitory factor (LIF)-signal transducer and activator of transcription 3 (STAT3) signaling. **f** Relative abundances of transcripts associated with cell proliferation. **g** Relative abundances of transcripts associated with transforming growth factor beta (TGFβ) signaling. Data are presented as mean ± standard error, *n* = 3. Means annotated with different letters are considered significantly different by one-way analysis of variance and Tukey’s honestly squared difference test, *P* < 0.05

To parse sets of genes whose expression in early canine reprogramming are sensitive to AA/RA supplementation or DMOG-mediated inhibition of 2-OG hydroxylases, we performed a more focused hierarchical clustering analysis to highlight specific relationships among gene expression patterns in 6 DPI samples (Fig. 5A). Cluster 1 identified transcripts induced by AA/RA exposure and partially abrogated by DMOG co-supplementation. Cluster 2 identified transcripts that were de-repressed in DMOG samples compared to AA/RA- or vehicle-treated transductants. Examples of gene products grouping into cluster 2 include those belonging to the TGFβ signaling pathway (ACVR1B, SMAD3, TGFB2, TGFBR1). Clusters 3 and 4 both identified transcripts that are suppressed by AA/RA treatment and generally insensitive to DMOG co-supplementation. Notably, clusters 3 and 4 were composed of WNT signaling pathway components (FZD7, CTNNB1, TCF3) and transcripts indicative of fibroblast-like cell populations (VIM, MSN, THY1, CD44).

Consistent with and building on our results for MET transcript panels, all epithelial markers profiled grouped in cluster 1. Both CDH1 (*P* = 4.09 × 10^−2^) and EPCAM (*P* = 1.84 × 10^−2^) were significantly elevated in AA/RA 6 DPI cultures compared to transductants co-supplemented with DMOG (Fig. 5B), whereas THY1 was significantly lower in the AA/RA group compared to both samples receiving DMOG/AA/RA or vehicle (Fig. 5B, *P* < 1.05 × 10^−3^). Interestingly, collections of transcripts also grouping into cluster 1 include pluripotency-associated transcription factors (NR0B1, ESRRB, KLF4, SALL4, NANOG), components of the LIF signaling pathway (LIFR, IL6ST, STAT3), and cell proliferation markers (CCNA2, CCNE1, CDC42, MYC). Among general markers of pluripotency, POU5F1 transcript abundance was significantly lower in the presence of DMOG (Fig. 5C, *P* < 1.29 × 10^−3^). Both NANOG and KLF4 transcript levels were significantly greater in 6 DPI transductants exposed to AA/RA compared to the vehicle group (Fig. 5C, *P* = 1.97 × 10^−4^ and *P* = 1.43 × 10^−2^). SOX2 and LIN28A expression were not affected by AA/RA, with or without DMOG, compared to 6 DPI intermediates receiving vehicle (Fig. 5C, *P* = 0.12). At this timepoint, both AA/RA and DMOG/AA/RA cultures exhibit lower expression of the enzyme ALPL (Fig. 5C, *P* = 2.17 × 10^−3^ and *P* = 2.70 × 10^−2^). Several transcription factors enriched in early embryonic cells and reflective of naïve transcriptional circuitry [63] are significantly more abundant in AA/RA transductants compared to 6 DPI cells treated with DMOG/AA/RA including ESRRB (*P* = 2.91 × 10^−2^), GBX2 (*P* = 1.04 × 10^−2^), KLF5 (*P* = 2.25 × 10^−2^), NR0B1 (*P* = 3.20 × 10^−2^), and SALL4 (Fig. 5D, *P* = 6.50 × 10^−6^). These findings are consistent with our expectation that the expression of a subset of pluripotency-associated transcription factors is facilitated by AA/RA treatment and at least partially abrogated by DMOG exposure.

Genes associated with the LIF signaling pathway such as LIFR (*P* = 5.03 × 10^−3^), IL6ST (*P* = 3.16 × 10^−2^), STAT3 (*P* = 6.52 × 10^−3^), and SOC3 (*P* = 4.51 × 10^−2^) were differentially expressed in early reprogramming intermediates supplemented with AA/RA compared to samples exposed to vehicle diluent or DMOG/AA/RA (Fig. 5E). Moreover, DMOG co-treatment was associated with decreased transcript levels for the proliferation-associated genes MYC (*P* = 1.81 × 10^−2^), CCNE1 (*P* = 2.21 × 10^−3^), CCNA2 (*P* = 1.32 × 10^−2^), and CDC42 (*P* = 8.13 × 10^−8^) but not MYCN (*P* = 0.69) when compared to cells receiving AA/RA alone (Fig. 5F). We also observed that multiple genes associated with TGFβ signaling including SMAD2 (*P* = 5.04 × 10^−3^), SMAD3 (*P* = 4.32 × 10^−3^), TGFB2 (*P* = 6.90 × 10^−4^), and TGFBR1 (*P* = 3.65 × 10^−3^) showed greater expression in samples allocated the DMOG/AA/RA treatment compared to the AA/RA condition (Fig. 5G). Overall, these results support the notion that 2-OG hydroxylase activity, either directly or indirectly, contributes to the regulation of cell proliferation as well as the LIF-STAT3 and TGFβ-SMAD2/3 signaling pathways in early canine reprogramming.

### Targeted expression profiling of canine embryonic stem cells and reprogramming intermediates distinguishes pluripotent cell-specific gene signatures

**Fig. 6:**
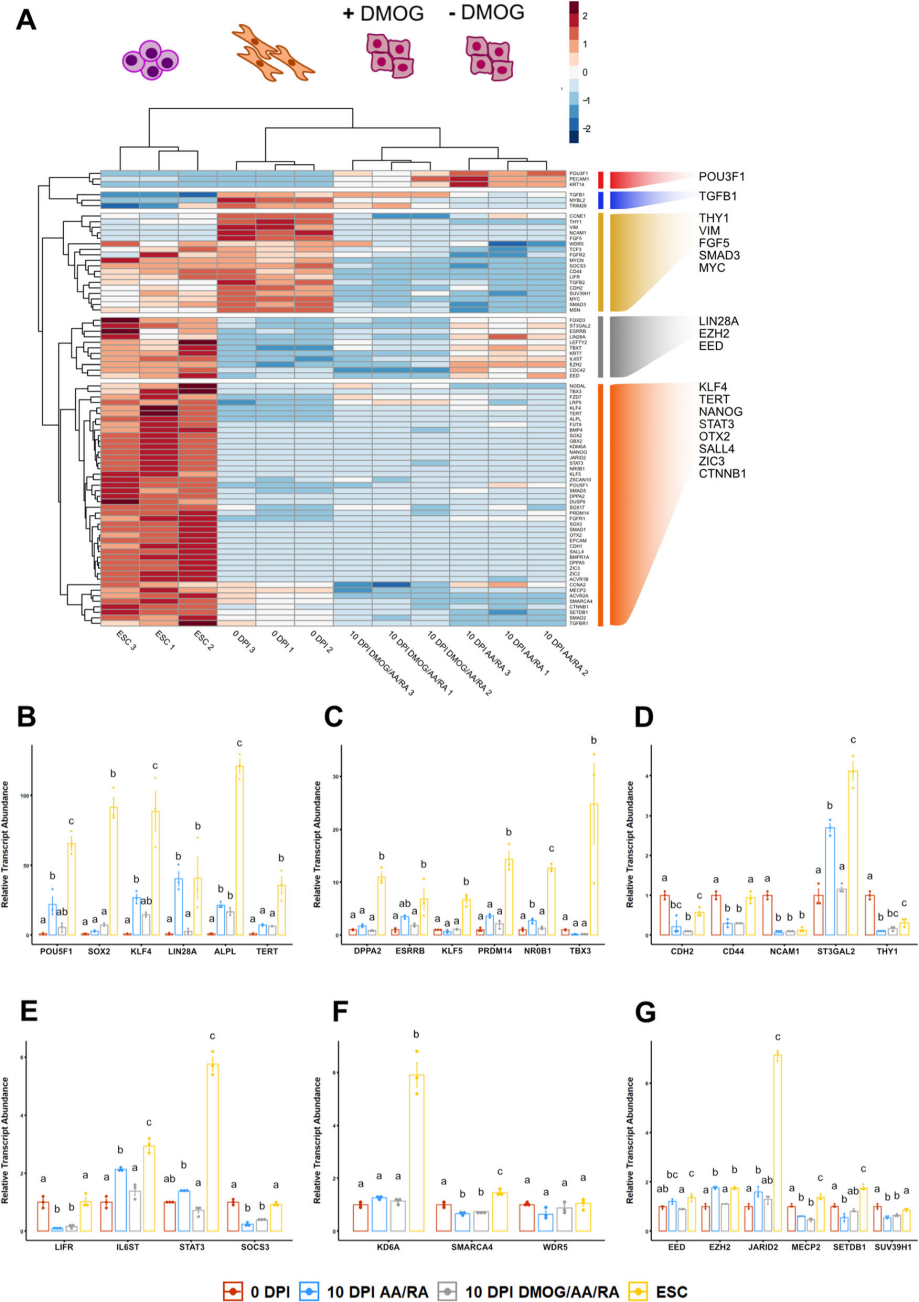
Primary colonies derived from transductants exposed to AA/RA exhibit few differentially expressed genes compared to DMOG/AA/RA colonies. **a** Hierarchical cluster analysis to identify subsets of genes that show specific expression patterns across specific samples of canine fetal fibroblasts (cFF; labeled 0 DPI), maturing (10 DPI) canine reprogramming intermediates, and cESC. Divergent color scale represents gene expression scaled by row. **b** Relative abundances of transcripts associated with cell pluripotency. **c** Relative abundances of transcripts associated with early embryonic (naïve) pluripotency. **d** Relative abundances of transcripts associated with cell surface proteins or the synthesis of cell surface antigens. **e** Relative abundances of transcripts associated with leukemia inhibitory factor (LIF)-signal transducer and activator of transcription 3 (STAT3) signaling. **f** Relative abundances of transcripts associated with chromatin-modifying or remodeling enzymes involved in the activation of gene transcription. **g** Relative abundances of transcripts associated with chromatin-modifying or remodeling enzymes involved in the repression of gene transcription. Data are presented as mean ± standard error, *n* = 3. Means annotated with different letters are considered significantly different by one-way analysis of variance and Tukey’s honestly squared difference test, *P* < 0.05

We next concentrated on the primary colonies derived from the AA/RA or DMOG/AA/RA treatment groups collected at 10 DPI and again used hierarchical clustering to assess relationships between gene expression patterns in the maturation phase of canine reprogramming (Fig. 6A). Cluster 1 included a small set of co-expressed genes enriched in 10 DPI reprogramming intermediates. Notably, the transcription factor POU3F1/OCT6, which is expressed in the epiblast and anterior neural plate [64], exhibited greater expression in maturing primary colonies compared to parental cFFs or cESCs (Supplemental Figure 3F, *P* < 3.02 × 10^−4^ and *P* < 4.67 × 10^−4^). Clusters 2 and 3 pinpointed genes associated with general cell proliferation and the fibroblast phenotype. Clusters 4 identified a large subset of transcripts enriched in cESCs that are also expressed in 10 DPI AA/RA colonies (FOXD3, ESRRB, LIN28A, TBXT), while genes grouping in cluster 5 exhibited an cESC-specific expression pattern (TBX3, KLF4, POU5F1, NANOG).

The primary colonies of reprogramming intermediates harvested at 10 DPI display similar expression patterns among the genes assessed in either the presence or absence of DMOG, with a few exceptions. Reprogramming intermediates from the 10 DPI AA/RA group showed significantly greater abundance of the LIN28A and NR0B1 transcripts compared to the 10 DPI DMOG/AA/RA colonies (Fig. 6B, C, *P* = 4.13 × 10^−2^ and *P* = 2.35 × 10^−2^). AA/RA colonies also showed a greater level of ST3GAL2 expression (Fig. 6D, *P* = 4.42 × 10^−4^), a sialyltransferase protein that produces the stage-specific embryonic antigen 4 (SSEA4) glycolipid [65]. Consistent with our findings in 6 DPI transductants, primary colonies that received DMOG treatment showed lower relative abundances of IL6ST and STAT3 transcripts (Fig. 6E, *P* = 8.13 × 10^−3^ and *P* = 2.55 × 10^−2^). Three enzymes involved with chromatin modifications or remodeling and linked to gene transactivation, specifically KDM6A (*P* = 0.97), SMARCA4/BRG1 (*P* = 0.48), and WDR5 (*P* = 0.41), did not significantly differ between AA/RA and DMOG/AA/RA colonies (Fig. 6F). Conversely, we observe DMOG supplementation is associated with decreased expression of the core polycomb repression complex 2 (PRC2)-associated proteins EED (*P* = 1.82 × 10^−2^) and EZH2 (*P* = 9.40 × 10^−6^) [66], compared to reprogramming intermediates treated with AA/RA alone (Fig. 6G). Collectively, these results indicate that transient exposure of canine reprogramming intermediates to AA/RA induces few latent changes in the expression of genes related to pluripotency, LIF signaling, and PRC2 components, which are mitigated by chemical antagonism of 2-OG hydroxylase activity.

**Fig. 7:**
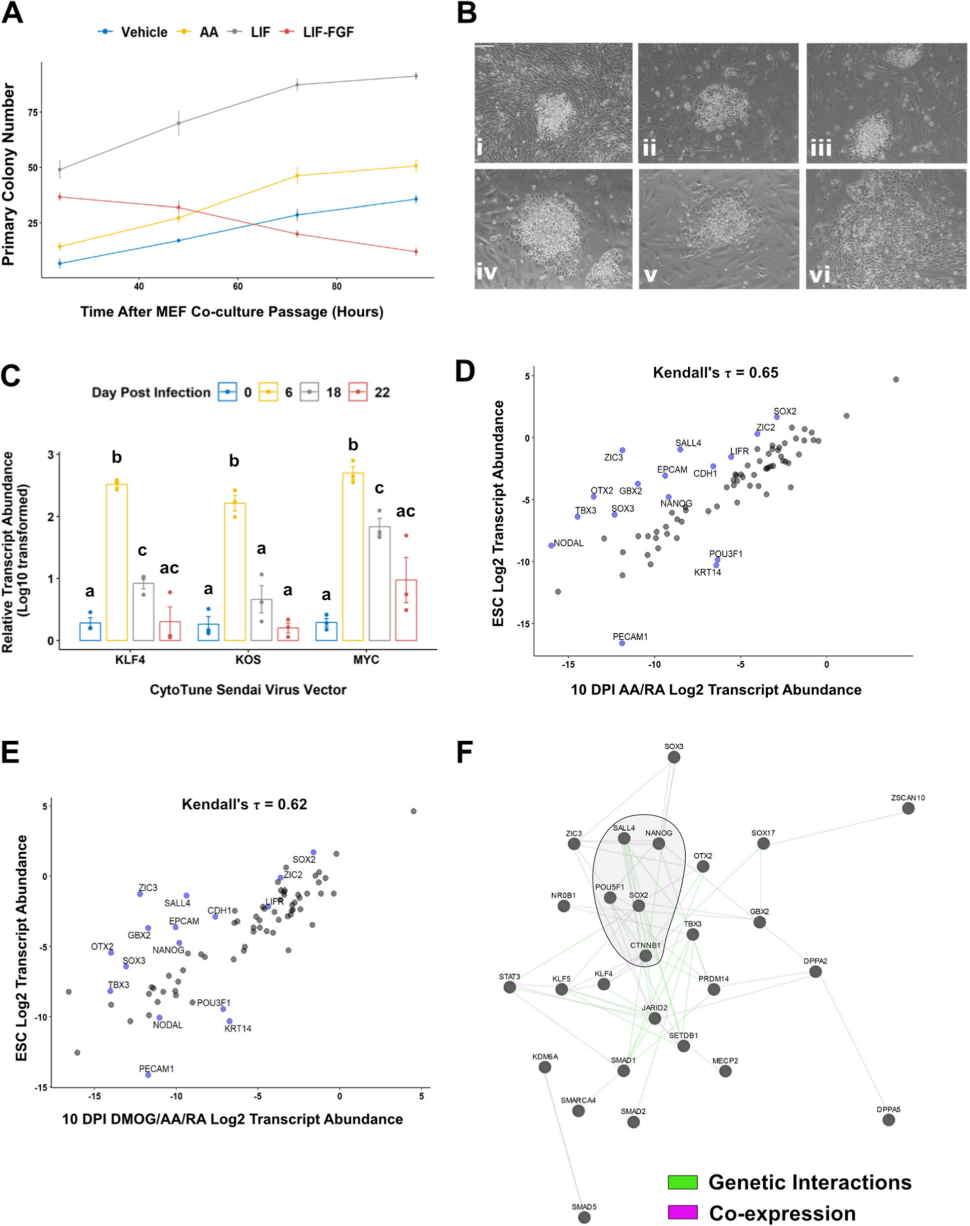
Canine reprogramming intermediates are depleted of a subset of pluripotency-associated factors expressed in cESC. **a** Summarization of raw counts of primary colonies emerging in the first 4 days after passaging transduced cFFs (7–10 DPI) in medium containing FBS. **b** Representative phase-contrast micrograph of one serially pick-passaged primary colony on (I) 10 DPI, (II) 14 DPI, (III) 18 DPI, (IV) 22 DPI, (V) 26 DPI, and (VI) 30 DPI. **c** Relative transcript abundance (Log10 transformed) of SeV-derived transcripts encoding KLF, KLF-OCT4-SOX2 (KOS), and MYC. Data are presented as mean ± standard error, *n* = 3. Means annotated with different letters are considered significantly different by one-way analysis of variance Tukey’s honestly squared difference test, *P* < 0.05. **d** Scatterplot of Log2-scaled expression ratios for a representative cESC sample versus a representative 10 DPI AA/RA sample. **e** Scatterplot of Log2-scaled expression ratios for a representative cESC sample versus a representative 10 DPI DMOG/AA/RA sample. Blue dots represent genes contributing to dissimilarity in the overall positive gene-gene association. **f** GeneMANIA network where nodes represent top differentially expressed genes in ESC versus 10 DPI primary colonies (AA/RA and DMOG/AA/RA groups). Edges represent known genetic interactions (green) or co-expression (purple) between genes based on datasets compiled by GeneMANIA. Densely interconnected nodes are shaded gray

To propagate maturing ciPSCs, we attempted single- and bulk-colony passaging techniques in different growth factor combinations (Fig. 7A). Culture medium composed of 10% FBS and DMEM was the most effective at facilitating primary colony formation between 7 and 10 DPI, which was accentuated with exogenous LIF supplementation (Fig. 7A). However, maturing ciPSCs progressively lost compact border morphology and gradually formed monolayer structures with radial asymmetry (Fig. 7B). Canine reprogramming intermediates could not be maintained beyond 3 weeks of reprogramming. At this time point, SeV-derived transcripts are not differentially abundant compared to non-transduced cFFs (Fig. 7C). Our differential gene expression analyses revealed that 20 of 24 pluripotency-associated transcription factors assessed by qPCR array displayed greater expression levels in cESCs when compared to either AA/RA or DMOG/AA/RA 10 DPI reprogramming intermediates (Fig. 6B, C and Supplemental Figure 3G-H, *P* < 0.05). We tested gene-gene correlations in cESCs and 10 DPI reprogramming intermediates to discern genes which do not have a common expression pattern (Fig. 7D, E). Comparisons of primary colonies exposed to AA/RA to cESC showed an overall positive correlation, but also that subsets of cell-cell adhesion proteins and transcription factors (e.g., SALL4, TBX3, SOX2) contribute to dissimilarity (Fig. 7D). Furthermore, this analysis underscored that disparities in gene expression between AA/RA-derived primary colonies and cESC samples were shared with cells from the DMOG/AA/RA condition (Fig. 7E). We reasoned that gene products involved in transcriptional regulation contribute to cell identity and derived a network from the 27 genes with DNA-binding or chromatin-binding functions and co-expressed in cESCs using GeneMANIA (Fig. 7F) [67]. The network analysis separated KDM6A and SMAD5 from a pluripotency sub-network consisting of transcription factors and epigenetic modifiers (Fig. 7F). Relatively dense interconnections between CTNNB1, SOX2, and SALL4 indicate that WNT/β-catenin signaling may have an important role in maintaining the pluripotent stem cell phenotype of cESCs (Fig. 7F). Together, these results suggest that 10 DPI reprogramming intermediates have not acquired a stable pluripotent state akin to cESCs. Furthermore, sustained re-expression of a network of transcriptional regulators that are enriched in cESCs during canine iPSC maturation may depend on alternative signaling pathways to LIF/STAT3.

## Discussion

**Fig. 8:**
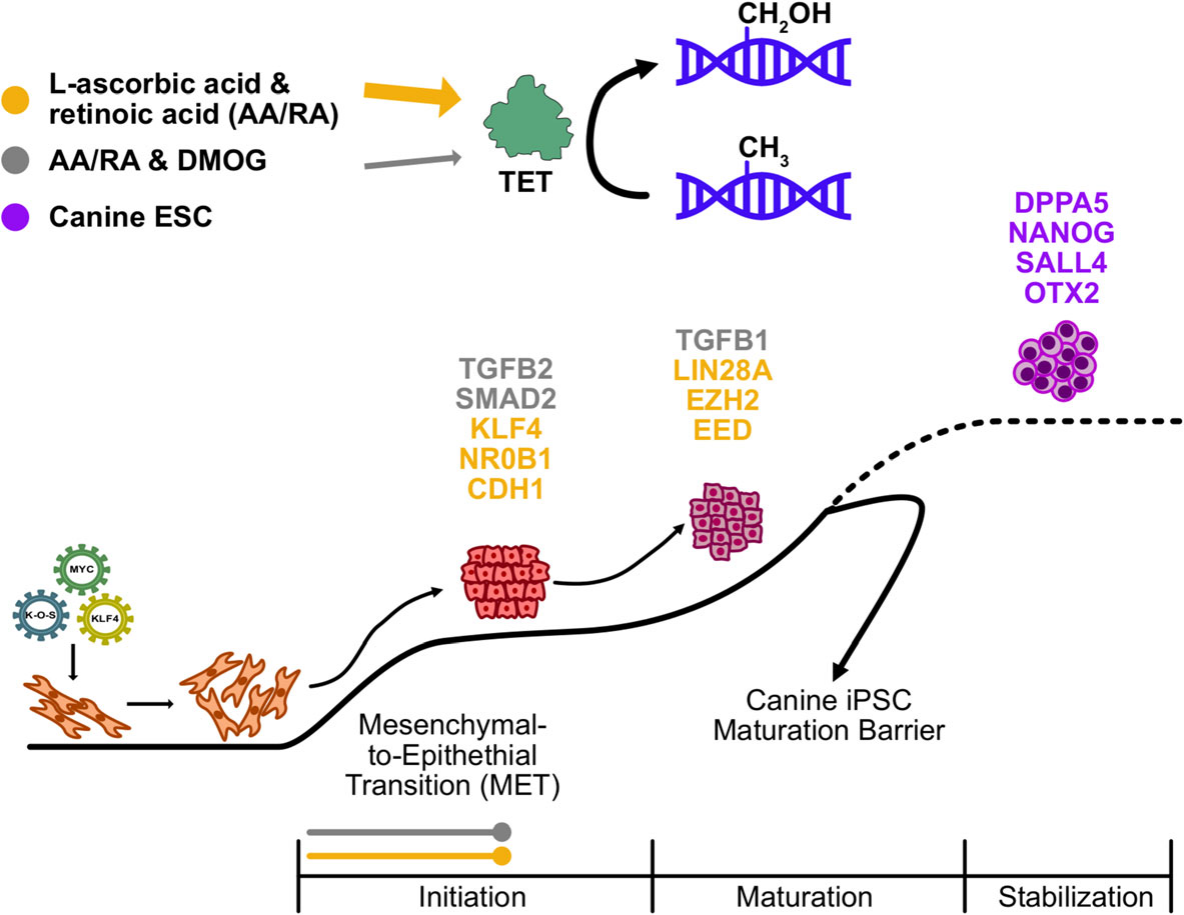
Schematic diagram of L-ascorbic acid and retinoic acid promoting the early reprogramming of canine fibroblasts. AA/RA L-ascorbic acid and retinoic acid, DMOG dimethyloxalylglycine, TET ten-eleven translocation dioxygenase, MYC myelocytomatosis proto-oncogene, KLF4 Krüppel-like factor 4, K-O-S KLF4-OCT4-SOX2

It is currently unknown if canine reprogramming mirrors the trajectory of human or mouse cells towards the iPSC state. Previous studies have focused on the molecular characteristics and differentiation potential of a limited number of putative canine iPSC clones derived from a single reprogramming experiment [16, 68]. These studies do not consider the discrete phases of somatic cell reprogramming and lacked a physiological pluripotent cell type, such as cESCs, to provide a reference transcriptional signature for a canine pluripotent stem cell. To improve the reproducibility of canine iPSC derivation, it is essential to identify potentially obstructive mechanisms to the reprogramming of canine cells by forced expression of transcription factors. Our targeted gene expression and DNA methylation assays indicate that partially reprogrammed cells derived from adult fibroblasts exhibit limited pluripotency factor expression, methylation of the NANOG promoter, and lower 5-hmC levels compared to cESC lines. We focused on gene regulation in early cFF reprogramming at timepoints with visible epithelial change among the bulk transductants and characterized a transcriptional response resembling MET. We determined that the initiation of cFF reprogramming, inferred from morphological and transcriptional features associated with MET, is amenable to reinforcement by AA/RA treatment, which modulate 5-hmC accumulation in canine fibroblasts. To our knowledge, this represents the first hypothesis-driven approach to improve the initiation of canine reprogramming with Sendai-based reprogramming vectors. Using SeV-transduced cFFs as a model to study early canine reprogramming, we identified reprogramming-associated genes responsive to AA/RA and/or DMOG, which indicate these loci may be regulated by 2-OG hydroxylases such as the TET paralogues. Lastly, we define a set of DNA- or chromatin-binding proteins from targeted gene expression profiling, which are depleted in reprogramming intermediates compared to pluripotent cESCs, as candidate positive regulators of canine somatic cell reprogramming (Fig. 8).

Comparisons of time-resolved data from reprogramming models using different cell types and/or species have identified context-specific trajectories and barriers to acquired pluripotency [69, 70]. We observed incomplete demethylation of the NANOG promoter region, and as a plausible consequence, endogenous NANOG transcripts were not upregulated in cPR clones. Limited endogenous pluripotency gene re-activation and constrained epigenetic remodeling is also evident in porcine reprogramming, a Laurasiatherian species like domestic dogs [71]. We found that endogenous POU5F1 expression in cPR lines does not correlate well with the methylation status of the − 394 to − 88 POU5F1 promoter region assessed in this study. However, high CpG content promoters like the canine POU5F1 locus may instead be under the regulator control of chromatin-modifying complexes [72]. Endogenous POU5F1 expression is indispensable for iPSC stabilization and maintenance of pluripotency [73]. Additionally, we made two key observations regarding the dynamics of endogenous POU5F1 expression in canine reprogramming. First, incompletely reprogrammed cPR lines stably re-express POU5F1 in a nuclear context where SOX2 and NANOG transcripts are not upregulated compared to donor cAF cultures. Second, POU5F1 transcript levels are lower in 6 DPI transductants exposed to 2-OG hydroxylase antagonist DMOG. Based on these findings, we posit that POU5F1 re-expression dynamics resemble the human reprogramming time course, wherein POU5F1 is reversibly re-expressed during MET, as opposed to during the later maturation phase in murine reprogramming [74, 75]. Species-specific mechanisms contributing to endogenous POU5F1 activation upon ectopic OSKM expression may be partly responsible for the different latencies observed in the reprogramming mouse (~ 20 days) or human (~ 30 days) somatic cells.

For nuclear reprogramming to occur during iPSC generation, thousands of loci gain or lose DNA methylation [19] as reprogramming factors direct active DNA demethylation mechanisms [31] and outcompete the somatic gene regulatory network to allow de novo DNA methylation of lineage-associated genes [76]. Higher reprogramming efficiencies between somatic cell types positively correlate with a number of hypermethylated tissue-specific genes shared with pluripotent ESCs [77]. Embryonic and fetal cell populations are often more efficiently reprogrammed, occasionally with fewer ectopic transcription factors expressed, compared to differentiated adult cells [78, 79]. We found no differences in the prevalence of 5-mC between adult or fetal canine fibroblasts, cPR cells, or cESCs. This finding is in line with observations that genome-wide methylation in the established pluripotent cells is similar to somatic cells unless small molecular inhibitors or activators are added to drive further epigenome erasure [80, 81]. However, genome-wide 5-hmC level was greater in cESCs and cPR cells which are considered more plastic than somatic fibroblasts. We detected greater expression levels of each mammalian TET paralogue in cFF compared to cAFs. In addition to dynamically regulating DNA methylation at regulatory elements, TET enzymes help maintain the hypomethylated state of high CpG density regions in pluripotent stem cells [82, 83]. TET1/Tet1 loss-of-function reduces reprogramming efficiency from both mouse and human somatic cells but is compatible with successful reprogramming due to functional compensation from TET2/3 [55, 84, 85]. In mouse reprogramming, *Tet1* is a direct target of exogenously expressed *Pou5f1* in early reprogramming (days 1–9) and reaches an expression level similar to mouse ESCs in late reprogramming (days 11–19) [55, 86]. Interestingly, TET2 appears the most important TET paralogue for human B cell reprogramming [31]. Similarly, we did not observe a significant induction of canine TET1 under standard reprogramming conditions lacking AA/RA. Moreover, experimental manipulations that increase TET abundance or catalytic activity promote 5-mC removal and iPSC generation in both human and murine cells [55, 87, 88]. Exogenous Tet1 expression has been reported to increase OSKM-mediated MEF initiation approximately 1.5-fold and could replace *Oct4* in the reprogramming workflow when combined with *Sox2*, *Klf4*, and *Myc* [55]. We observe a 2.7-fold increase in the rate of primary colony induction accompanied by the activation of an early subset of pluripotency genes when cFFs transduced with human OSKM are exposed to AA/RA. However, without reporter-linked genes to faithfully track reprogramming progress, we expect colony enumeration to be less discriminative for bona fide canine iPSC emergence. L-ascorbic acid has been shown to directly interact with the TET catalytic domain as a cofactor [34, 35] and enhances Fe(II) recycling [37] to elevate 5-mC hydroxylation activity, whereas retinoic acid is proposed to synergistically increase TET action via nuclear receptor complex-dependent functions (RAR/RXR) such as upregulation of TET2 and TET3 expression [37] and/or focal recruitment of TETs and base-excision repair machinery [36]. Retinoid ligands also have TET-independent mechanisms through NR5A2/LRH1 and RAR-gamma (RARG) signaling to regulate early human and mouse reprogramming as well as the induction of naïve pluripotency-associated genes [59, 89].

The first phase of cell reprogramming is initiation, which encompasses several of transgene-dependent events including hyper-proliferation and loss of somatic cell identity [53]. The specific cellular and molecular events that define the initiation phase is dependent on cell type [70] and in fibroblast reprogramming is highlighted by MET [20, 90]. In our single-infection reprogramming system, we observe altered expression of a variety of growth factors, adhesion molecules, cytoskeleton components, and transcriptional regulators at 6 DPI, which were consistent with the process of MET. Interestingly, exposure to AA/RA enhanced several previously established aspects of MET including a switch from neuronal-type to epithelial-type cadherin expression [91] and the induction of cytokeratin gene expression [92]. In addition to markers of MET, several transcripts associated with pluripotency, LIF signaling, and cell proliferation are sensitive to AA/RA and DMOG. While a certain level of biological variability and technical noise is expected from these types of comparisons, the observed changes in mRNA abundances are consistent with the observed cellular phenotypes and are sensitive to experimental activation or inhibition of 2-oxoglutarate hydroxylases. Our interpretation of these findings is that these pathways and factors are regulated at the transcriptional level by 2-OG hydroxylase enzymes in early transcription factor-mediated reprogramming of cFFs. At the time this study was underway, there were no commercially available chemical inhibitors selective for TET catalytic activity, and therefore, we are not able to rule out the contribution of prolyl hydroxylase enzymes or the Jumonji-C family of lysine demethylases [58]. Transcriptional perturbations to TET paralogues in combination by TET1/2/3 triple knockdowns in future investigations will clarify the specific contributions of this family of epigenetic modifiers. The sampling and analysis of heterogeneous cell populations is another caveat of the study as the influence of specific subsets of canine reprogramming intermediates remains unknown. Cell surface proteins associated with pluripotency are often divergent between species, as observed for CD90/THY1, CD40, and CD30 when tracking mouse or human reprogramming, thereby necessitating the empirical identification of these surface markers [76, 93]. As the early transcriptional response to OSKM reprogramming implicates several gene products localized to the cell surface (CDH1, LIFR, EPCAM), fluorescence-activated cell sorting and enrichment of cell populations with epithelial or mesenchymal characteristics will deconvolute the pathways and processes active in productively reprogramming versus refractory cell populations. Still, observations from bulk transductants in reprogramming experiments have proven insightful regarding the specific barriers to reprogramming [57, 90], and the molecular signatures of intermediate reprogramming states have been further refined with single-cell approaches [94]. Other reprogramming vector systems may yield results that differ from our observations because the trajectory of cell states towards pluripotency and overall kinetics of iPSC generation is influenced by the reprogramming system utilized [95]. For instance, heterogeneity in expression of SeV-derived OSKM is expected to cause differences between the reprogramming intermediates being studied. Another explanation is that the transcript markers selected in our assays are biased towards genes with a known role in pluripotency induction and therefore do not reveal other changes in the global transcriptome resulting from Sendai reprogramming vector transduction.

Although pluripotency-associated transcription factors are well-conserved at the amino acid level in vertebrates, analyses of gene expression patterns and transcription factor binding sites have revealed extensive rewiring of transcriptional regulatory interactions [96–98]. Alterations to hierarchical relationships between existing TFs can lead to divergence in the expression patterns of orthologous genes and may contribute to species-specific determinants in development and reprogramming [96, 99]. Our strategy to stabilize maturing canine iPSC intermediates in culture media used for routine culture of canine ESC and putative iPSC was unsuccessful beyond days 26–30 of reprogramming. Fundamental questions remain outstanding regarding the trophic requirements and regulatory processes that achieve a transgene-independent pluripotent state in the dog. Defining the culture conditions that support the maturation and prolonged self-renewal of pluripotent cells from dogs remains an ongoing challenge. We and others have expanded cESC [45] and ciPSC [15, 100] in culture medium formulations intended and optimized for human ESC, but we have observed deficiencies in the glycolysis pathway of cESCs cultured in this medium [101]. To date, canine reprogramming studies have only applied small molecule inhibitors to facilitate reprogramming and species-specific determinants of iPSC derivation are challenging to predict [14]. Herein, we discerned a subset of factors involved in gene regulation that are selectively enriched in cESCs compared to actively reprogramming intermediates using a targeted qPCR profiling assay. We found that the pluripotency-associated factors DPPA5, NANOG, NR0B1, ZIC3, SALL4, OTX2, GBX2, and TBX3 distinguish cESCs from primary colonies at reprogramming day 10. Ectopic expression of these candidate TFs in the OSKM reprogramming workflow is a logical extension of this finding which may reveal nuclear determinants sufficient for canine iPSC maturation. Interestingly, SALL4, POU5F1, and OTX2 were differentially expressed in a microarray study by Chow et al. comparing a candidate canine iPSC line to iPSC-derived mesenchymal stem cells [16]. The knowledge of cESC-enriched transcripts that are depleted in reprogramming intermediates will inform new testable hypotheses for future studies concentrating on canine iPSC maturation and stabilization.

## Conclusions

Our study represents the first investigation to identify hurdles specific to discrete phases of canine induced pluripotency. Our examination of early canine reprogramming suggests that MET initiates 4 to 6 days after cFF transductions with the OSKM transcription factors. We conclude that 2-OG hydroxylases contribute to the effect of AA/RA culture medium supplementation in early canine reprogramming by facilitating the transcriptional activation of a subset of pluripotency factors and developmental signaling pathway genes. As the barriers to obtaining canine iPSC remain poorly described, our finding that simple adaptations to reprogramming media promote the loss of somatic identity and primary colony formation is a significant step towards the development of reproducible reprogramming in canine donor cells. This study also lends support to an evolutionarily conserved TET regulatory mechanism controlling the MET program, which has broader implications in the areas of developmental and cancer biology.

## Supplementary Information

**Additional file 1: Supplemental Figure 1.** Canine adult fibroblasts transduced with Sendai virus vectors encoding OSKM generate partially reprogrammed cell lines. (A) Representative phase-contrast and fluorescent micrographs of a partially reprogrammed clonal cell population (cPR-L6) staining positive for stage-specific embryonic antigen 4 (SSEA4). (B) Relative transcript abundance of core pluripotency factors (OCT4, SOX2, NANOG) and tri-lineage markers (BRACHYURY, GATA6, NESTIN) in partially reprogrammed (cPR) cell lines and parental adult fibroblast (cAF), *n* = 3. (C) Dot plots depict unmethylated (open circle) and methylated (filled circle) CpG dinucleotides fragments at POU5F1 and NANOG loci in seven representative technical replicates. Mean adjusted methylation level for whole promoter fragments at canine (D) NANOG and (E) POU5F1/OCT4 loci, *n* = 4. Data are presented as mean ± standard error. Means annotated with different letters are considered significantly different by one-way analysis of variance and Tukey’s honestly squared difference test, *P* < 0.05.

**Additional file 2: Supplemental Figure 2.** Maturity of donor tissue influences primary canine fibroblast proliferation and the effect of L-ascorbic acid or retinoic acid on TET paralogue transcript level. (A) Isotype controls for direct immunofluorescence staining of canine fetal fibroblast (cFF) cultures, related to (i) CD44-PE or (ii) CD90-PE staining experiments in Fig. 1A. (B) Fold-increase in total cell number over five days of adherent canine adult fibroblasts (cAF) and cFFs. (C) Population doubling time intervals calculated from linear growth phase in cAF and cFF. (D) Ratio of 5-hydroxymethylcytosine (5-hmC) to 5-methylcytosine (5-mC) in media containing AA/RA or vehicle diluent. Data are presented as mean ± standard error, n = 4. Relative transcript abundances for (E) cTET1, (F) cTET2 and (G) cTET3 in canine dermal fibroblasts cultured in atmospheric oxygen and exposed to various concentrations of AA and/or RA, *n* = 3. (H) Representative phase-contrast and fluorescent micrographs of canine dermal fibroblast transduced with emGFP control Sendai vector at various multiplicity of infection (MOI). (I) Summarization of the percent emGFP-positive cells at each MOI determined by flow cytometry. Data are presented as mean *n* = 2. Means annotated with different letters are considered significantly different by one-way or two-way analysis of variance and Tukey’s honestly squared difference test, P < 0.05.

**Additional file 3: Supplemental Figure 3.** Effect of dimethyloxalylglycine (DMOG) dose escalation on canine fetal fibroblasts and top-ranking transcripts by component loading and *p*-values. (A) Population doubling time intervals calculated from linear growth phase after 96 h of DMOG treatment. (B) Percent 5-hydroxymethylcytosine calculated after 96 h of DMOG treatment. (C) Relative ALPL transcript abundance in parental cFF cells (0 DPI), bulk transductants at 6 DPI, 10 DPI primary colonies or canine embryonic stem cells (ESC). (D) Alkaline phosphatase staining visualized by transmitted light microscopy in (I) bulk transductants evidently without colony formation, (II) primary colonies expressing endogenous pluripotency genes by RT-qPCR, or (III) canine ESC colonies cultured on mouse embryonic fibroblasts (MEFs). (E) PCA factor loadings plot displaying the top 2% of transcripts contributing to sample variance. (F) Maturation barplot for primed pluripotency genes. (G) Ranked p-value (−Log_10_ transformed) barplot for AA/RA versus cESC. (H) Ranked p-value (−Log_10_ transformed) barplot for DMOG/AA/RA versus cESC. Data are presented as mean ± standard error, n = 3. Means annotated with different letters are considered significantly different by one-way analysis of variance and Tukey’s honestly squared difference test, P < 0.05.

**Additional file 4: Supplemental Table 1.** Oligonucleotide primers used for RT-qPCR. **Supplemental Table 2.** Oligonucleotide primers used for bisulfite PCR. *Primer sequences obtained from [15]. **Supplemental Table 3.** Transcript targets for custom RT2 Profiler qPCR Array.

**Additional file 5.** Metadata and normalized values

## Data Availability

Metadata and normalized expression ratios generated from canine reprogramming RT^2^ Profiler PCR Array experiments in this study are included in Supplemental Data File 1.

## Abbreviations

iPSC: Induced pluripotent stem cell
OSKM: OCT4 SOX2 KLF4 MYC
LIF: Leukemia inhibitory factor
FGF2: Fibroblast growth factor 2
TET: Ten-eleven translocation
2-OG: 2-Oxoglutarate
5-mC: 5-Methylcytosine
5-hmC: 5-Hydroxymethylcytosine
MEF: Mouse embryonic fibroblast
MET: Mesenchymal-to-epithelial transition
cESC: Canine embryonic stem cell
FBS: Fetal bovine serum
SeV: Sendai virus
MOI: Multiplicity of infection
AA: L-ascorbic acid
RA: Retinoic acid
CpG: Cytosine-phosphate-guanine
cPR: Canine partially reprogrammed cell
cAF: Canine adult fibroblast
cFF: Canine fetal fibroblast
DMOG: Dimethyloxalylglycine
SSEA4: Stage-specific embryonic antigen 4
PRC2: Polycomb repressive complex 2
