# Supplementary figures and images for: Targeted expression profiling reveals distinct stages of early canine fibroblast reprogramming are regulated by 2-oxoglutarate hydroxylases
Source: Stem Cell Res Ther. 2020 Dec 9;11:528. doi: 10.1186/s13287-020-02047-1 (PMC7725121; doi:10.1186/s13287-020-02047-1)

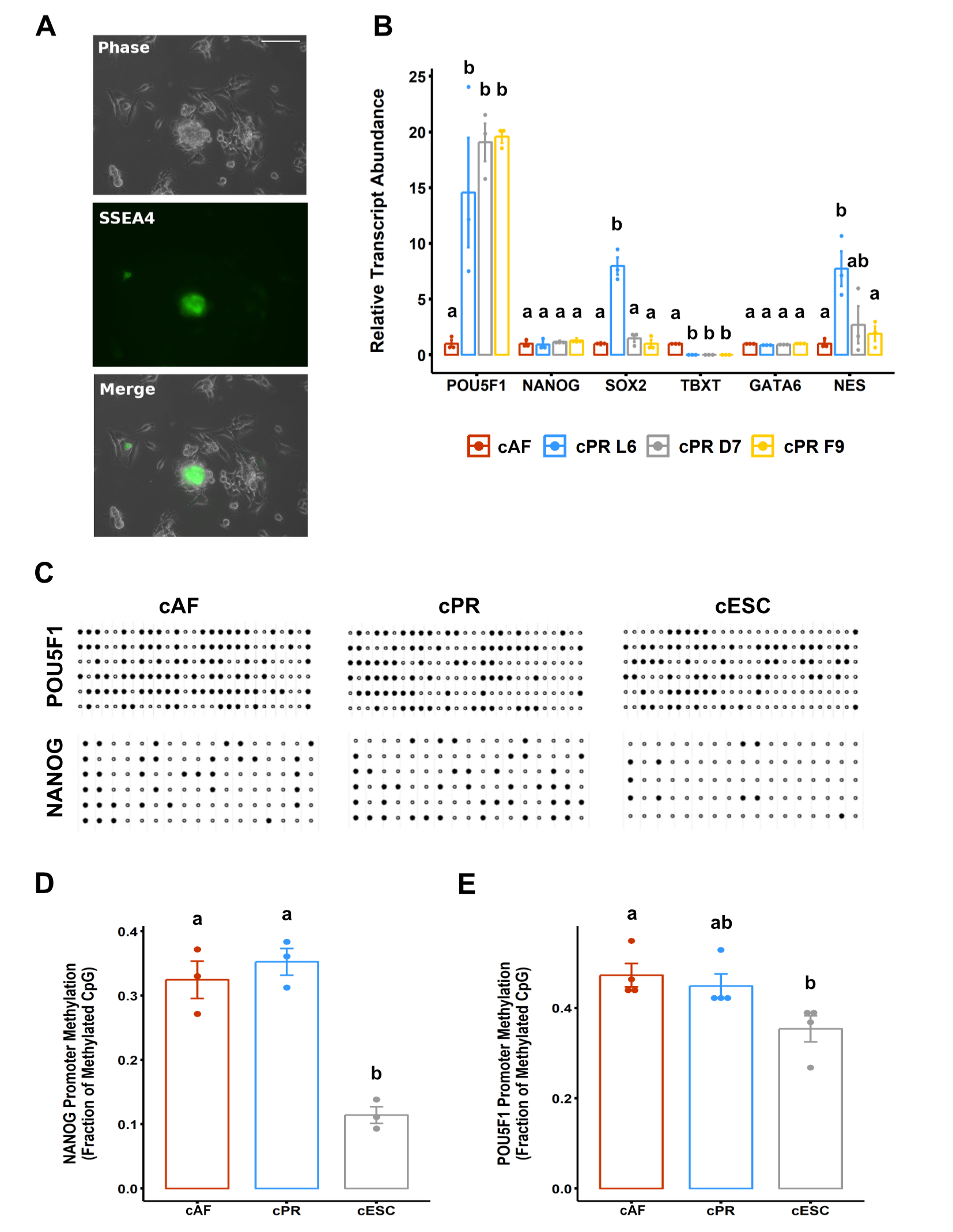

Supplement: Supplementary file 1 — Additional file 1: Supplemental Figure 1. Canine adult fibroblasts transduced with Sendai virus vectors encoding OSKM generate partially reprogrammed cell lines. (A) Representative phase-contrast and fluorescent micrographs of a partially reprogrammed clonal cell population (cPR-L6) staining positive for stage-specific embryonic antigen 4 (SSEA4). (B) Relative transcript abundance of core pluripotency factors (OCT4, SOX2, NANOG) and tri-lineage markers (BRACHYURY, GATA6, NESTIN) in partially reprogrammed (cPR) cell lines and parental adult fibroblast (cAF), n = 3. (C) Dot plots depict unmethylated (open circle) and methylated (filled circle) CpG dinucleotides fragments at POU5F1 and NANOG loci in seven representative technical replicates. Mean adjusted methylation level for whole promoter fragments at canine (D) NANOG and (E) POU5F1/OCT4 loci, n = 4. Data are presented as mean ± standard error. Means annotated with different letters are considered significantly different by one-way analysis of variance and Tukey’s honestly squared difference test, P < 0.05. [file 13287_2020_2047_MOESM1_ESM.tiff]

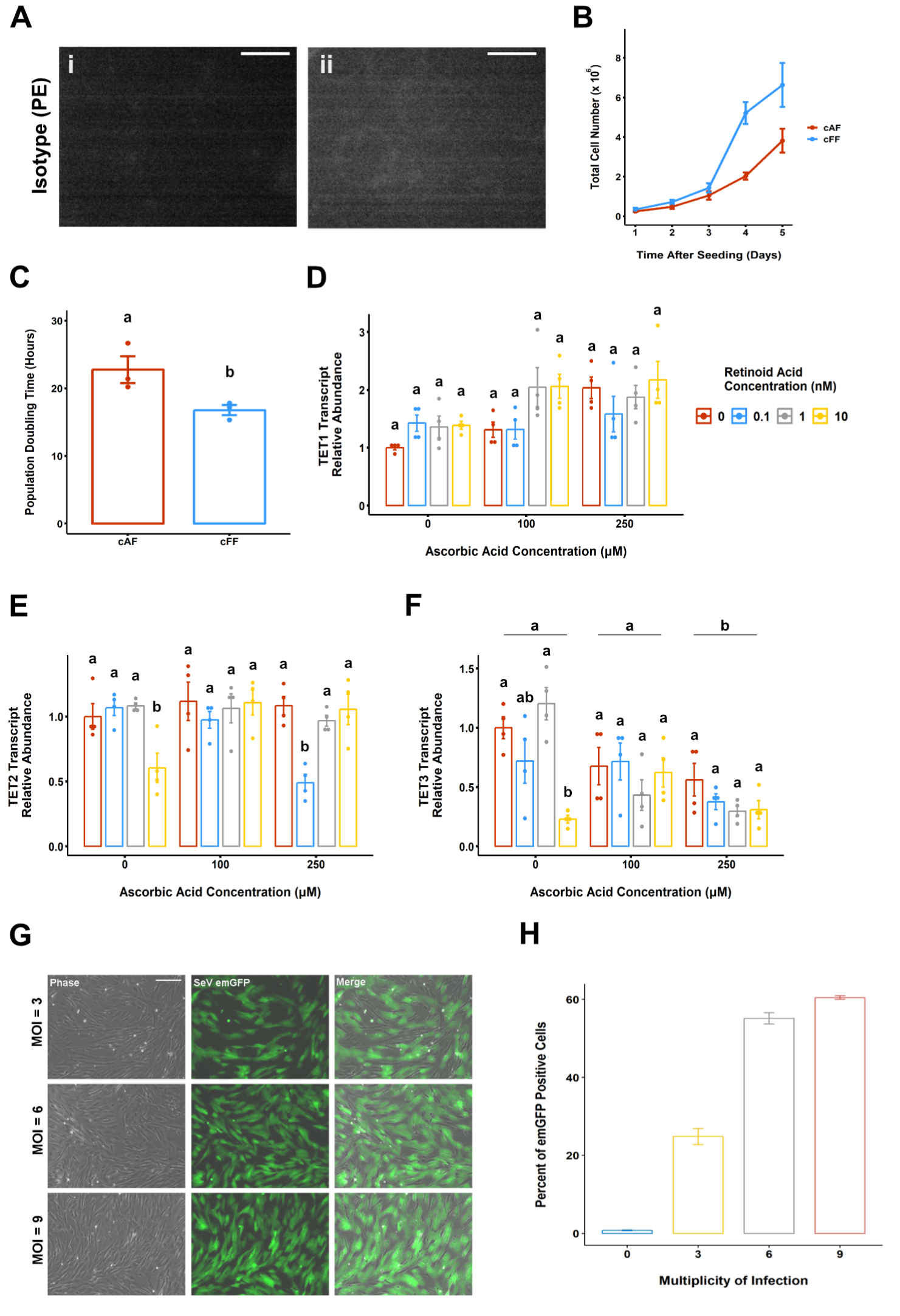

Supplement: Supplementary file 2 — Additional file 2: Supplemental Figure 2. Maturity of donor tissue influences primary canine fibroblast proliferation and the effect of L-ascorbic acid or retinoic acid on TET paralogue transcript level. (A) Isotype controls for direct immunofluorescence staining of canine fetal fibroblast (cFF) cultures, related to (i) CD44-PE or (ii) CD90-PE staining experiments in Fig. 1A. (B) Fold-increase in total cell number over five days of adherent canine adult fibroblasts (cAF) and cFFs. (C) Population doubling time intervals calculated from linear growth phase in cAF and cFF. (D) Ratio of 5-hydroxymethylcytosine (5-hmC) to 5-methylcytosine (5-mC) in media containing AA/RA or vehicle diluent. Data are presented as mean ± standard error, n = 4. Relative transcript abundances for (E) cTET1, (F) cTET2 and (G) cTET3 in canine dermal fibroblasts cultured in atmospheric oxygen and exposed to various concentrations of AA and/or RA, n = 3. (H) Representative phase-contrast and fluorescent micrographs of canine dermal fibroblast transduced with emGFP control Sendai vector at various multiplicity of infection (MOI). (I) Summarization of the percent emGFP-positive cells at each MOI determined by flow cytometry. Data are presented as mean n = 2. Means annotated with different letters are considered significantly different by one-way or two-way analysis of variance and Tukey’s honestly squared difference test, P < 0.05. [file 13287_2020_2047_MOESM2_ESM.tiff]

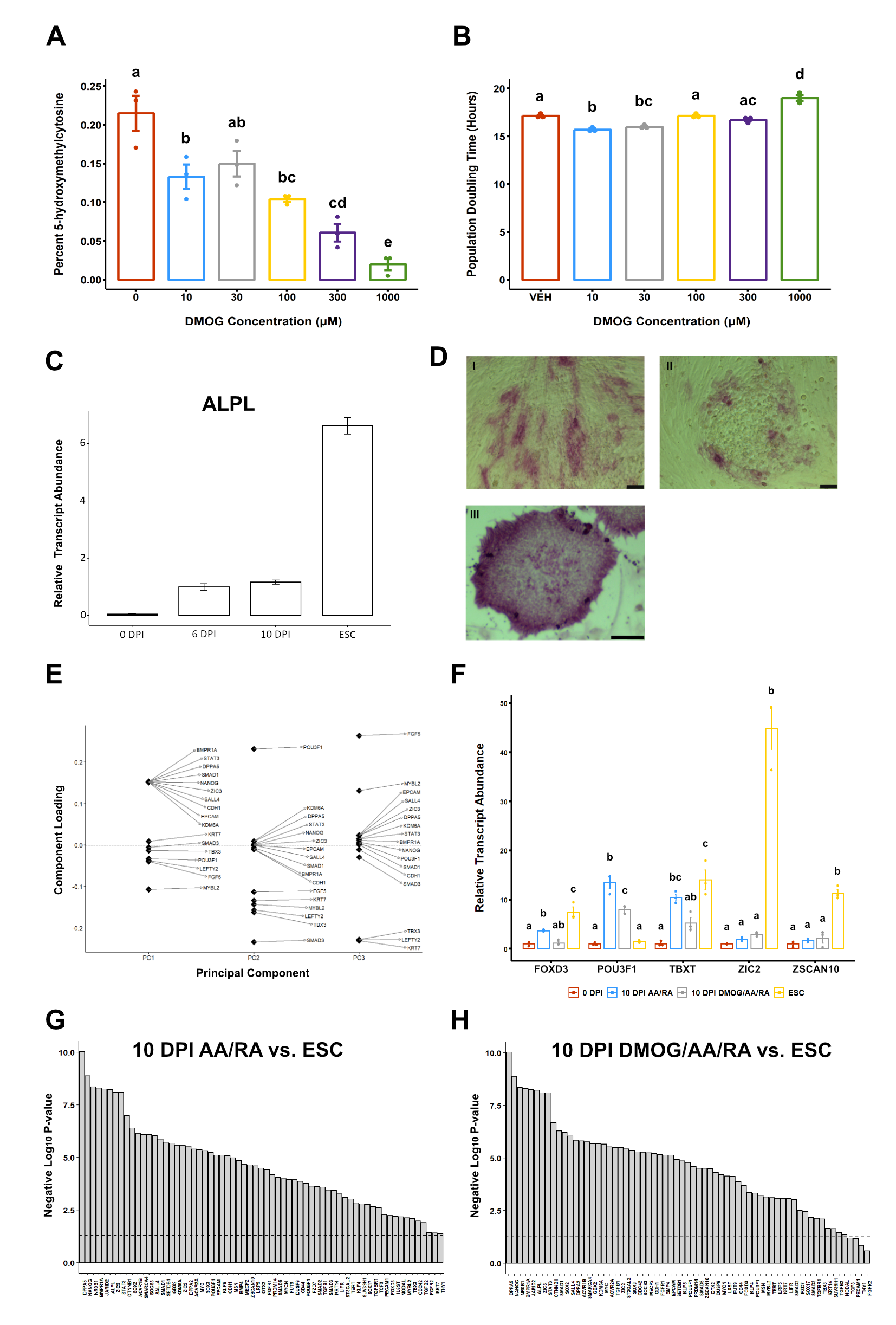

Supplement: Supplementary file 3 — Additional file 3: Supplemental Figure 3. Effect of dimethyloxalylglycine (DMOG) dose escalation on canine fetal fibroblasts and top-ranking transcripts by component loading and p-values. (A) Population doubling time intervals calculated from linear growth phase after 96 h of DMOG treatment. (B) Percent 5-hydroxymethylcytosine calculated after 96 h of DMOG treatment. (C) Relative ALPL transcript abundance in parental cFF cells (0 DPI), bulk transductants at 6 DPI, 10 DPI primary colonies or canine embryonic stem cells (ESC). (D) Alkaline phosphatase staining visualized by transmitted light microscopy in (I) bulk transductants evidently without colony formation, (II) primary colonies expressing endogenous pluripotency genes by RT-qPCR, or (III) canine ESC colonies cultured on mouse embryonic fibroblasts (MEFs). (E) PCA factor loadings plot displaying the top 2% of transcripts contributing to sample variance. (F) Maturation barplot for primed pluripotency genes. (G) Ranked p-value (−Log10 transformed) barplot for AA/RA versus cESC. (H) Ranked p-value (−Log10 transformed) barplot for DMOG/AA/RA versus cESC. Data are presented as mean ± standard error, n = 3. Means annotated with different letters are considered significantly different by one-way analysis of variance and Tukey’s honestly squared difference test, P < 0.05. [file 13287_2020_2047_MOESM3_ESM.tiff]
