# Supplementary material for: Targeted expression profiling reveals distinct stages of early canine fibroblast reprogramming are regulated by 2-oxoglutarate hydroxylases
Source: Stem Cell Res Ther. 2020 Dec 9;11:528. doi: 10.1186/s13287-020-02047-1 (PMC7725121; doi:10.1186/s13287-020-02047-1)
Supplement: Supplementary file 4 — Additional file 4: Supplemental Table 1. Oligonucleotide primers used for RT-qPCR. Supplemental Table 2. Oligonucleotide primers used for bisulfite PCR. *Primer sequences obtained from [15]. Supplemental Table 3. Transcript targets for custom RT2 Profiler qPCR Array. [file 13287_2020_2047_MOESM4_ESM.docx]

**Supplemental Tables**

**Supplemental Table 1. Oligonucleotide primers used for RT-qPCR**

| **Target** | **Forward** | **Reverse** | **Accession** | **Tm** |
| --- | --- | --- | --- | --- |
| TET1 | CCAGTTAACCCGACCTGTCC | CGGCCGAGAGCCATTAGAAA | XM_022417496.1 | 60 |
| TET2 | AGCACATTGGTATGCACCCT | TGTCGGCATGTCTTGACTGG | XM_535678.6 | 60 |
| TET3 | GCCCCCTACTCAGGAAATGA | GGTAGAAAAAGGGCTCTCGGG | XM_005630554.3 | 62 |
| TBP | GAGCTCTGGGATCGTGCCGC | TCAGTGCAGTGGTTCGGGGC | XM_005627735.3 | 60 |
| RPS5 | TCACTGGTGAGAACCCCCT | CCTGATTCACACGGCGTAG | XM_533568 | 60 |

**Supplemental Table 2. Oligonucleotide primers used for bisulfite PCR**

| **Target** | **Forward** | **Reverse** | **Accession** | **Tm** |
| --- | --- | --- | --- | --- |
| Nanog OUT* | GTATTTTTGATTTTAAAGGATGGA | AAAACCTCCACATATAAAAAATAAA | NC_006609.3 | 59 |
| Nanog IN* | TAGAAATATTTAATTGTGGGGTT | CATATAAAAAATAAAAAAAAACAAAAT | NC_006609.3 | 60 |
| OCT4 OUT* | AGGTTAGTGGGTGGGATTGG | CCTTAAAACAACAACCCCACTC | NC_006594.3 | 64 |
| OCT4 IN* | AGGTGTTGAGTAGTTTTTAGGAGA | ACTCCCACCTAAAATCCACAATA | NC_006594.3 | 62 |

*Primer sequences obtained from (15).

**Supplemental Table 3. Transcript targets for custom RT2 Profiler qPCR Array**

| **Gene Symbol** | **Alias** | **Full Gene Name** |
| --- | --- | --- |
| POU5F1 | OCT4 | POU Class 5 Homeobox 1 |
| SOX2 | SOX2 | SRY box Transcription Factor 2 |
| KLF4 | KLF4 | Krüppel like Factor 4 |
| NANOG | NANOG | Nanog Homeobox |
| ESRRB | ERR2 | Estrogen Related Receptor Beta |
| PRDM14 | PFM11 | PR/SET Domain 14 |
| OTX2 | OTX2 | Orthodenticle Homeobox 2 |
| LIN28A | LIN28A | Lin-28 Homolog A |
| TBXT | T | T box Transcription Factor |
| SOX3 | SOX3 | SRY box Transcription Factor 3 |
| POU3F1 | OCT6 | POU Class 3 Homeobox 1 |
| MYC | MYCC | MYC Proto-oncogene |
| MYCN | MYCN | MYCN Proto-oncogene |
| CCNE1 | CCNE1 | Cyclin E1 |
| CDC42 | CDC42 | Cell Division Cycle 42 |
| CCNA2 | CCNA2 | Cyclin A2 |
| ALPL | TNAP | Alkaline Phosphatase |
| TERT | TERT | Telomerase Reverse Transcriptase |
| MYBL2 | BMYB | MYB Proto-oncogene like 2 |
| TRIM28 | KAP1 | Tripartite Motif Containing 28 |
| TGFB2 | TGFB2 | Transforming Growth Factor Beta 2 |
| TGFB1 | TGFB1 | Transforming Growth Factor Beta 1 |
| BMP4 | BMP4 | Bone Morphogenetic Protein 4 |
| NODAL | NODAL | Nodal Growth Differentiation Factor |
| SMAD1 | SMAD1 | SMAD Family Member 1 |
| SMAD2 | SMAD2 | SMAD Family Member 2 |
| SMAD3 | SMAD3 | SMAD Family Member 3 |
| CTNNB1 | CTNNB1 | Catenin Beta 1 |
| TCF3 | E2A | Transcription Factor 3 |
| STAT3 | STAT3 | Signal Transducer and Activator of Transcription 2 |
| FGFR1 | FGFR1 | Fibroblast Growth Factor Receptor 1 |
| PECAM1 | CD31 | Platelet and Endothelial Cell Adhesion Molecule 1 |
| CD44 | ECMR-III | CD44 Molecule |
| THY1 | CD90 | Thy-1 Cell Surface Antigen |
| EPCAM | CD326 | Epithelial Cell Adhesion Molecule |
| CDH1 | ECAD | Cadherin 1 (Epithelial) |
| CDH2 | NCAD | Cadherin 2 (Neuronal) |
| NCAM1 | CD56 | Neuronal Cell Adhesion Molecule 1 |
| KRT14 | CK14 | Keratin 14 |
| KRT7 | CK7 | Keratin 7 |
| VIM | VIM | Vimentin |
| MSN | MSN | Moesin |
| NR5A2 | LRH1 | Nuclear Receptor Subfamily 5 Group A Member 2 |
| NR0B1 | DAX1 | Nuclear Receptor Subfamily 0 Group B Member 1 |
| TBX3 | TBX3 | T box Transcription Factor 3 |
| DPPA5 | DPPA5 | Developmental Pluripotency Associated 5 |
| SALL4 | SALL4 | Spalt like Transcription Factor 4 |
| DPPA2 | DPPA2 | Developmental Pluripotency Associated 2 |
| GBX2 | GBX2 | Gastrulation Brain Homeobox2 |
| KLF5 | KLF5 | Krüppel like Factor 5 |
| SOX17 | SOX17 | SRY box Transcription Factor 17 |
| FGF5 | FGF5 | Fibroblast Growth Factor 5 |
| FOXD3 | FOXD3 | Forkhead box D3 |
| ZSCAN10 | ZNF206 | Zinc Finger and SCAN Domain Containing 10 |
| ZIC2 | ZIC2 | Zic Family Member 2 |
| ZIC3 | ZNF203 | Zic Family Member 3 |
| LIFR | CD118 | Leukemia Inhibitor Factor Receptor Subunit Alpha |
| IL6ST | CD130 | Interleukin 6 Signal Transducer |
| BMPR1A | CD292 | Bone Morphogenetic Protein Receptor Type 1A |
| SMAD5 | SMAD5 | SMAD Family Member 5 |
| SOCS3 | SOCS3 | Suppressor of Cytokine Signaling 3 |
| LEFTY2 | LEFTA | Left-Right Determination Factor 2 |
| ACVR2A | ACVR2A | Activin Receptor Type 2A |
| ACVR1B | ACVR1B | Activin Receptor Type 1B |
| TGFBR1 | ALK5 | Transforming Growth Factor Beta Receptor 1 |
| FGFR2 | FGFR2 | Fibroblast Growth Factor Receptor 2 |
| DUSP6 | MKP3 | Dual Specificity Phosphatase 6 |
| FZD7 | FZD7 | Frizzled Class Receptor 7 |
| LRP5 | LRP5 | LDL Receptor Related Protein 5 |
| EZH2 | EZH2 | Enhancer of Zeste Homologue 2 |
| JARID2 | JARID2 | Jumonji and AT-rich Interaction Domain Containing 2 |
| WDR5 | WDR5 | WD Repeat Domain 5 |
| SMARCA4 | BRG1 | SWI/SNF Related, Matrix-associated, Actin-dependent regulator of chromatin, Subfamily A Member 4 |
| KDM6A | UTX | Lysine Demethylase 6A |
| SUV39H1 | KMT1A | Suppressor of Variegation 3-9 Homolog 1 |
| SETDB1 | KMT1E | SET Domain Bifurcated Histone Lysine Methyltransferase 1 |
| MECP2 | MECP2 | Methyl CpG Binding Protein 2 |
| EED | EED | Embryonic Ectoderm Development |
| FUT9 | FUT9 | Fucosyltransferase 9 |
| ST3GAL2 | ST3GAL2 | ST3 Beta-Galactoside Alpha-2,3-Sialyltransferase 2 |
